# Multi-environment Genomic Selection in Rice Elite Breeding Lines

**DOI:** 10.1186/s12284-023-00623-6

**Published:** 2023-02-08

**Authors:** Van Hieu Nguyen, Rose Imee Zhella Morantte, Vitaliano Lopena, Holden Verdeprado, Rosemary Murori, Alexis Ndayiragije, Sanjay Kumar Katiyar, Md Rafiqul Islam, Roselyne Uside Juma, Hayde Flandez-Galvez, Jean-Christophe Glaszmann, Joshua N. Cobb, Jérôme Bartholomé

**Affiliations:** 1 CIRAD, UMR AGAP Institut, 34398 Montpellier, France; 2 UMR AGAP Institut, Univ Montpellier, CIRAD, INRAE, Institut Agro, Montpellier, France; 3 Rice Breeding Innovation Platform, International Rice Research Institute, DAPO, Box7777, Metro Manila, Philippines; 4 Institute of Crop Science, College of Agriculture and Food Science, University of the Philippines, Los Baños, Laguna Philippines; 5 RiceTec. Inc, PO Box 1305, Alvin, TX 77512 USA; 6 CIRAD, UMR AGAP Institut, Cali, Colombia; 7 Alliance Bioversity-CIAT, Cali, Colombia

**Keywords:** Rice, *Oryza sativa*, Elite lines, Genomic prediction, Genotype by environment interactions, Environmental covariates, Multi-environment genomic prediction models

## Abstract

**Background:**

Assessing the performance of elite lines in target environments is essential for breeding programs to select the most relevant genotypes. One of the main complexities in this task resides in accounting for the genotype by environment interactions. Genomic prediction models that integrate information from multi-environment trials and environmental covariates can be efficient tools in this context. The objective of this study was to assess the predictive ability of different genomic prediction models to optimize the use of multi-environment information. We used 111 elite breeding lines representing the diversity of the international rice research institute breeding program for irrigated ecosystems. The lines were evaluated for three traits (days to flowering, plant height, and grain yield) in 15 environments in Asia and Africa and genotyped with 882 SNP markers. We evaluated the efficiency of genomic prediction to predict untested environments using seven multi-environment models and three cross-validation scenarios.

**Results:**

The elite lines were found to belong to the *indica* group and more specifically the *indica-1B* subgroup which gathered improved material originating from the Green Revolution. Phenotypic correlations between environments were high for days to flowering and plant height (33% and 54% of pairwise correlation greater than 0.5) but low for grain yield (lower than 0.2 in most cases). Clustering analyses based on environmental covariates separated Asia’s and Africa's environments into different clusters or subclusters. The predictive abilities ranged from 0.06 to 0.79 for days to flowering, 0.25–0.88 for plant height, and − 0.29–0.62 for grain yield. We found that models integrating genotype-by-environment interaction effects did not perform significantly better than models integrating only main effects (genotypes and environment or environmental covariates). The different cross-validation scenarios showed that, in most cases, the use of all available environments gave better results than a subset.

**Conclusion:**

Multi-environment genomic prediction models with main effects were sufficient for accurate phenotypic prediction of elite lines in targeted environments. These results will help refine the testing strategy to update the genomic prediction models to improve predictive ability.

**Supplementary Information:**

The online version contains supplementary material available at 10.1186/s12284-023-00623-6.

## Introduction

Rice (*Oryza sativa* L.) is one of the most important food crops in the world and in Asia in particular. About 3.5 billion people depend on rice as their main food source. As the world's population increases, the demand for rice will be under pressure as an estimated 116 million additional tons of rice will be needed to meet demand by 2035 (Seck et al. 2012). In this context, genetic improvement for yield potential is considered to be one of the most effective strategies to meet this growing demand and also to address the growing impact of climate change on rice production (Saito et al. 2021). Rice breeders, therefore, must increase yield potential at a greater pace (Cobb et al. 2019). However, the use of conventional breeding methods is time-consuming and can take up to ten years to develop and evaluate new elite varieties (Collard and Mackill 2008). To some extent, the advances of marker-assisted selection (MAS) enable faster development of new varieties but are limited to the introgression favorable alleles of major genes or quantitative trait loci (QTLs) with large effects mainly related to abiotic (e.g. submergence, salinity) or biotic (e.g. blast, bacterial leaf blight) stress tolerance into elite backgrounds (Gregorio et al. 2013; Jena and Mackill 2008). MAS is not tailored to enhance the effectiveness of breeding strategies for quantitative traits like grain yield which are governed by a large number of genes or QTLs with small effects (Jena and Mackill 2008).

With the reduction in genotyping costs, genomic selection (GS) has arisen as a more efficient option for breeding program optimization (Ahmadi et al. 2020; Heffner et al. 2009). GS can accelerate the rate of genetic gain without significantly increasing the size of the breeding program by reducing the length of the breeding cycle (Cobb et al. 2019). GS uses genome-wide markers (mainly SNPs markers) to predict the genomic estimated breeding values (GEBV) of selection candidates based on statistical models trained on a reference population that is both genotyped and phenotyped (Ahmadi et al. 2020; Jannink et al. 2010; Meuwissen et al. 2001). Since 2010, many GS studies have been published on small grain crops such as wheat, barley, oats, or rice, indicating that GS has been successfully applied in cereals breeding programs to increase the rate of genetic gain (Crossa et al. 2017). More recently, genomic prediction models integrating multi-environment data have emerged in the plant breeding community in order to increase accuracy by modeling the genotype-by-environment interactions (G × E) rather than ignoring them (Burgueño et al. 2012; Heslot et al. 2013; Jarquín et al. 2014; Lopez-Cruz et al. 2015). The G × E interactions in plant breeding are usually evaluated through multi-environment trials and refer to changes in the ranking of genotypes between environments (Freeman 1973). The G × E analysis also plays a key role in evaluating the stability of genotypes across environments (Cooper et al. 1993; Elias et al. 2016). Crossa et al. (2022) have recently reviewed the evolution of genomic prediction models that consider G × E interactions. Burgueño et al. (2012) and Schulz-Streeck et al. (2013) proposed the first multi-environment prediction models. These models were subsequently enhanced by using different statistical regressions and kernel methods (Crossa et al. 2019; Cuevas et al. 2016, 2019; Lopez-Cruz et al. 2015, p.; Montesinos et al. 2016, 2018), or by using crop growth models (Cooper 2015; Heslot et al. 2014; Messina et al. 2017; Rincent et al. 2017) and recently by using reaction-norm models integrating the information of environmental covariates, such as weather and soil information of the experimental trials, for prediction in the context of G × E (Costa-Neto et al. 2020, 2021; de los Campos et al. 2020; Jarquín et al. 2014; Ly et al. 2018; Millet et al. 2019; Morais Júnior et al. 2017). In this latter approach, G × E is accounted for by using the interaction between markers and environmental covariates (ECs) and has been shown to increase the accuracy of genomic prediction in plant breeding. For example, Jarquín et al. (2014), using wheat data, reported an increase in the accuracy of the reaction-norm model integrating ECs compared to models with main effects alone. The effectiveness of the use of ECs in GS is also discussed in the literature (Costa-Neto et al. 2020; Heslot et al. 2014; Millet et al. 2019; Monteverde et al. 2019; Morais Júnior et al. 2017). In rice, a large number of GS studies have been published since 2014, when the first empirically based study was published (see a review by Bartholomé et al. 2022). Through these studies, we gained a better understanding of the benefits and limitations of GS in the context of rice breeding. The impact of trait architecture, population structure, the training set size, and composition, as well as marker density, has been well covered. However, the impact of G × E has received somewhat less attention. Indeed, only a few studies using breeding material have used multi-environment models including G × E (Ben Hassen et al. 2018; Monteverde et al. 2018, 2019; Morais Júnior et al. 2017). The conclusions arising from these works based on a relatively small number of environments are that multi-environment models tend to give higher prediction accuracies.

This study aimed to assess the efficiency of multi-environment genomic prediction models in the context of an applied breeding program. We used an elite core panel that represents the elite diversity managed by the irrigated rice breeding program at the International Rice Research Institute (IRRI). This panel was phenotyped in 15 environments in Asia and Africa regions from 2018 to 2020. This information from multi-environment trials (phenotypic data and environmental covariates) was used to characterize the level of G × E interaction and to cluster the environments. We then compared seven genomic prediction models to evaluate the impact of modeling G × E and environmental covariates on predictive abilities when new environments were predicted.

## Materials and Methods

### Plant Material and Genotypic Characterization

The plant material consisted of 111 elite lines from the IRRI breeding program for irrigated systems (Additional file 1: Table S1), hereafter referred to as the elite core panel (ECP). The ECP represents the elite diversity of the parental lines used in IRRI’s breeding program for irrigated systems and is derived mostly from the breeding efforts that were conducted at IRRI since the 1960s (Juma et al. 2021). The population included recent varieties such as IRRI 154, IRRI 156, IRRI 174, IRRI 180, IRRI 186, and IRRI 193 as well as current parental lines.

The ECP was genotyped using the 1K Rice Custom Amplicon assay (1K-RiCA, Arbelaez et al. 2019). Leaf tissues of single plants of each line of the ECP were collected and freeze-dried. Genomic DNA (gDNA) was extracted using the CTAB method (Cetyl Trimethyl Ammonium Bromide), as described by Murray and Thompson (1980). The quality of gDNA was visually checked on 1% agarose gel. The quantity of gDNA was then evaluated using PicoGreen^®^ (https://www.biotek.com) fluorometric kits and adjusted to obtain a concentration close to 10 ng/µl gDNA for the library preparation. Illumina^®^’s TruSeq Custom Amplicon chemistry was used to create the libraries and the sequencing was performed using the MiSeq Sequencing-by-Synthesis Technology System. A custom SNP-calling pipeline was used to align sequence data on the Nipponbare rice genome MSU7 (Kawahara et al. 2013). The sequences with non-alignment and multiple positions were then removed. SNP data was saved in a HapMap format (Gibbs and et al. 2003). The raw SNP data was then filtered with TASSEL 5 (Bradbury et al. 2007). The SNPs with more than 20% of missing data, a minor allele frequency (MAF) lower than 5%, and a percentage of heterozygous calls greater than 10 were removed. Consequently, four out of 111 ECP lines have been removed from the list. A final set of 107 lines and 882 SNP markers distributed along the rice genome was used for the analyses (Additional file 2: Fig. S1). The genotypic information for the ECP is available in HapMap format (Additional file 3).

The genotypic characterization of the ECP in relation to *O. sativa* subgroups was performed by combining the genotypic data of the ECP from the 1K-RiCA assay above with the 3000 rice genomes (3 K-RG) data (Wang et al. 2018). The physical positions of the 882 SNPs were used to extract a dataset of filtered SNPs for the entire 3 K-RG using the rice SNP-seek database (Mansueto et al. 2017). As a result, a total of 837 SNPs in common in both data sets were used for downstream analysis. The SNPs were then encoded from nucleotide alleles into numeric genotypes as 0, 0.5, and 1. A principal component analysis (PCA) was carried out using the function *dudi.pca* within the R package *ade4* (Dray and Dufour 2007), the PCs were then visualized using the R package *ggplot2* (Wickham 2016). An unweighted neighbor-joining tree between ECP and 3 K-RG’s subgroups was constructed using TASSEL 5 software (Bradbury et al. 2007).

### Multi-environment Evaluation of the Elite Breeding Lines

Within the IRRI breeding program framework, the ECP was evaluated in multi-environment trials at 12 different locations including IRRI headquarter (Los Baños, the Philippines) and research stations from partners in Asia and Africa. The information regarding the locations of the 15 field experiments is available in Table 1 and Additional file 1: Table S2. The experiments were carried out in both the dry (DS) and wet seasons (WS) from 2018 to 2020. Different experimental designs were used to accommodate partners' capacities: alpha-lattice, randomized complete block, row-column, partially replicated, or systematic arrangement designs with either one or two replicates for each. Due to limited seed availability, not all the elite lines were evaluated in all 15 experiments resulting in sparse testing evaluation. The number of lines evaluated in each location ranged from 39 to 111 lines, as detailed in Table 1 and Additional file 2: Fig. S2. Most of the experiments were carried out by transplanting, except for one experiment conducted with direct seeding (at Maputo–Mozambique). Standard management practices were applied in all trials with basal fertilizer applications along with chemical and/or manual pest and weed control.

**Table 1:** Information of fifteen yield trials conducted on the elite core panel (ECP)

Country	Location	Year & Season	Study name	No. lines	Experimental design	Replication level	No. checks	Seeding date	Harvest date
Bangladesh	Gazipur	2019-Wet	BD-GZ-19W	93 (90)	P-REP	27% lines	9	2019-07-08	2019-11-05
Bangladesh	Nizmawna	2019-Wet	BD-NM-19W	93 (90)	Systematic arrangement	1	6	2019-07-11	2019-11-10
India	Hyderabad	2018-Wet	IN-HY-18W	39 (37)	RCBD	2	4	2018-07-17	2018-11-27
India	Cuttack	2019-Wet	IN-CU-19W	40 (38)	RCBD	2	11	2019-07-10	2019-11-19
India	Hyderabad	2019-Dry	IN-HY-19D	39 (37)	RCBD	2	4	2019-01-17	2019-05-19
India	Hyderabad	2019-Wet	IN-HY-19W	40 (38)	Augmented RCBD	3% lines	5	2019-07-02	2019-12-07
India	Maruteru	2019-Wet	IN-MA-19W	40 (38)	P-REP	43% lines	4	2019-07-06	2019-11-17
India	Raipur	2019-Wet	IN-RP-19W	40 (38)	P-REP	43% lines	5	2019-07-15	2019-12-03
Kenya	Ahero	2019-Dry	KE-AH-19D	92 (89)	RCBD	2	5	2019-08-21	2020-01-04
Kenya	Mwea	2020-Wet	KE-MW-20W	92 (89)	RCBD	2	5	2020-02-24	2020-07-19
Mozambique	Chokwe	2020-Wet	MZ-CK-20W	93 (90)	Row-Column	2	3	2019-11-05	2020-04-09
Mozambique	Maputo	2020-Wet	MZ-MP-20W	93 (90)	Row-Column	2	3	2019-11-21	2020-04-11
Philippines	Los Baños	2019-Dry	PH-LB-19D	111 (107)	Alpha Lattice	2	5	2019-01-15	2019-05-13
Philippines	Los Baños	2019-Wet	PH-LB-19W	111 (107)	Alpha Lattice	2	5	2019-06-20	2019-10-17
Tanzania	Dakawa	2020-Wet	TZ-DK-20W	91 (88)	Augmented RCBD	5% lines	6	2020-03-16	2020-08-01

At the No. lines column, the numbers contained within the brackets show the numbers of lines having SNP data from the 1K-RiCA dataset

Three agronomic traits were measured on each elite line: days to flowering (DTF), plant height (HT), and grain yield (YLD). DTF (days) were calculated as the number of days from seeding to the time of 50% of the plants flowering within a plot. The plant height (cm) was measured from the ground level to the tip of the highest panicle (awns excluded) at the maturity of five randomly selected plants for each elite line. For grain yield (tons/ha), each plot was harvested excluding border rows. From this sample, grain moisture content was measured using a moisture meter. Then, plot-level grain yield was computed as the grain weight in kilograms from each plot, normalized at 14% of moisture, and adjusted by the harvested areas to obtain the yield in tons per hectare.

### Phenotypic Data Analysis

For the statistical analysis of the trials, two linear mixed models were used to take account of the diversity of the experimental design. The general form of the base models was:

$$y = Xb + Zu + e$$

where $y$ is the vector of phenotypes, $b$ is the vector of fixed effects, and $X$ is the associated design matrix, $u$ is the vector of random effects and $Z$ is the associated design matrix, and $e$ is the vector of residuals. For trials with a rectangular field layout, a model with first-order autoregressive spatial structure (AR1 ⊗ AR1) was used (Gilmour et al. 1997). For these models, all vectors and incidence matrices are the same as the base model above, it only differs in the structure of variance residuals. The matrices of variance residuals are defined as R = σ^2^_e_Σc(pc) ⊗ Σr(pr), where σ^2^_e_ is the variance components of residual, Σc(pc) and Σr(pr) are the correlation matrices of the first-order autoregressive, pc and pr are the autocorrelation parameters for the spatial coordinates, columns, and rows of plots respectively, ⊗ is the Kronecker product from the auto-regressive process in columns and rows, respectively. The factors of fixed, random, and residual effects for statistical models of each trial were described in detail in Additional file 1: Table S3.

The analyses were performed using the *asreml()* function of the R package *asreml* (version 4.1.0.143) (Butler et al. 2017)*.* Broad-sense heritability (H^2^) was estimated for each trait using the following formula:

$$H^{2} = \sigma_{g}^{2} / \left( {\sigma_{g}^{2} + \sigma_{e}^{2} } \right)$$

where σ^2^_g_ is the genotypic variance obtained from the experimental data and *σ*^*2*^_*e*_ is the residual variance obtained from the model. H^2^ and the associated standard error were estimated with the function *predict()*. The best linear unbiased predictors (BLUPs) for all genotypes were extracted for each trial and each trait and were used as adjusted phenotypes for further analysis. For the two trials without the replications (Nizmawna-Bangladesh and Hyderabad-India), we used the phenotypic data directly. The phenotypic information of the ECP is available in Additional file 4.

We considered an environment as the combination of location, year, and season. Analysis of correlation between the three traits within single environments, and between environments was performed using the Pearson correlation method within the *ggpairs()* function in the *GGally* R package (Schloerke and et al. 2020). The hierarchical clustering analysis of the environments was carried out using the *pvclust* package in R (Suzuki and Shimodaira 2006).

A simple analysis of variance (ANOVA) for genotype by environment interaction (G × E) upon the phenotypic performance of ECP was also carried out using the *metan* R package (Olivoto and Lúcio 2020).

### Weather Data and Environmental Covariates

The weather data of each environment were obtained from the NASA POWER database (https://power.larc.nasa.gov/). This database was queried using the R package *nasapower* (Sparks 2018) via the R packages *EnvRtype* (Costa-Neto et al. 2021). The *get_weather* function was used to retrieve daily weather data based on the geographical coordinates (N latitude and E longitude) of each trial. The following daily weather variable from the transplanting date to the harvesting date was obtained for all the trials: the total precipitation (PP, mm), the dew-point temperature at two meters (DPT, °C d^−1^), the minimum, maximum and mean temperature at two meters (TMIN, TMAX and TM, °C d^−1^), the relative humidity at two meters (RH, %), the all-sky surface photosynthetically active radiation total (APAR, W m^2^) and the clear sky surface photosynthetically active radiation total (CPAR, W m^2^). The *processWTH* function from the R package *EnvRtype* was then used to compute the temperature range (TR, °C), the potential evapotranspiration (PET, mm d^−1^) and the vapor pressure deficit (VPD, kPa d^−1^) (Costa-Neto et al. 2021). Finally, eight environmental covariates (ECs) were selected for further analysis: PP, DPT, PET, VPD, TM, TR, APAR, and CPAR (Additional file 1: Table S4).

To assess the effects of ECs through different developmental phases of the ECP on the genomic predictive ability, the phenology of the crop was identified for each environment consisting of: the vegetative phase (from the transplanting date to the earliest line); the reproductive phase (the interval between the earliest and latest date of flowering); and the ripening phase (from the latest date of flowering up to the latest harvest date) (Additional file 2: Fig. S3). The information on the 24-ECs is available in Additional file 5. The evaluation of the level of similarity between environments (based on ECs) was performed by the hierarchical clustering analysis using the *pvclust* package of R (Suzuki and Shimodaira 2006).

### Genomic Prediction Analysis

#### Statistical Models for Genomic Prediction Analysis

Due to its stability of accuracy across different environments and traits and its ease of implementation, GBLUP (genomic best linear unbiased prediction) is the most used method on rice (Bartholomé et al. 2022). In this study, we focused our effort on GBLUP, which is currently used routinely at IRRI. Seven genomic prediction models were implemented to predict DTF, HT, and YLD. The first model was the standard GBLUP model with only the main effect of the genotypes (VanRaden 2008):

1 $${\text{Model}}\;{\text{G}}:\;y_{i} = \mu + g_{i} + \varepsilon_{i}$$

where μ is the overall mean; $g_{i}$ is the random effect of the i-th genotype, denoted as $g_{{}} \sim N\left( {0, \sigma_{g}^{2} G_{{}} } \right)$ with the genomic relationship matrix (G) estimated as G = X * X^T^/*p*, in which X is the *n* × *p* matrix of centered and standardized markers, n is the number of genotypes and *p* is the number of markers and $\varepsilon_{i}$ is the residual effects denoted as $\varepsilon_{{}} \sim N\left( {0, \sigma_{\varepsilon }^{2} } \right)$. The G model was used as a baseline model to construct the remaining six models by adding the main effect of the environments (E), the environmental covariates (W), or the interaction effects with G (G × E and GxW) into the model (1). Ultimately, four models included only the main effects and three models also included interaction terms based on the approach of reaction norm models developed by (Jarquín et al. 2014):

2 $${\text{Model}}\;{\text{GE}}:y_{ij} = \mu + g_{i} + e_{j} + \varepsilon_{ij}$$

3 $${\text{Model}}\;{\text{GW}}:y_{ij} = \mu + g_{i} + w_{ij} + \varepsilon_{ij}$$

4 $${\text{Model}}\;{\text{GEW}}:y_{ij} = \mu + g_{i} + e_{j} + w_{ij} + \varepsilon_{ij}$$

5 $${\text{Model}}\;{\text{GE}} - {\text{G}} \times {\text{E}}:y_{ij} = \mu + g_{i} + e_{j} + ge_{ij} + \varepsilon_{ij}$$

6 $${\text{Model}}\;{\text{GW}} - {\text{G}} \times {\text{W}}:y_{ij} = \mu + g_{i} + w_{ij} + gw_{ij} + \varepsilon_{ij}$$

7 $${\text{Model}}\;{\text{GEW}} - {\text{G}} \times {\text{E}} - {\text{G}} \times {\text{W}}:y_{ij} = \mu + g_{i} + e_{j} + w_{ij} + ge_{ij} + gw_{ij} + \varepsilon_{ij}$$

where *e*_*j*_ is the effect of the *j-th* environment which is denoted as *e* $\sim$ N(0, $\sigma_{e}^{2}$), with $\sigma_{e}^{2}$ representing the variance component of the environments; *ge*_*ij*_ is the interaction effects of the *i-th* genotypic within the *j-th* environment which is modeled by the Hadamard product of $Z_{g} GZ_{g}^{T}$ and $Z_{e} Z_{e}^{T}$, denoted as $ge \sim N\left( {0, \left[ {Z_{g} GZ_{g}^{T} } \right] \circ \left[ {Z_{e} Z_{e}^{T} } \right]\sigma_{ge}^{2} } \right)$ with Z_e_ as the incidence matrix for the environmental effects that connect the phenotypes with environments; *w*_*ij*_ is the effect of the environmental covariates (ECs) in the *ij-th* genotype X environment combination which is denoted as $w \sim N\left( {0, \Omega \sigma_{w}^{2} } \right)$ with Ω computed using ECs and proportional to WW’, where W is a matrix with centred and standardised values of the ECs*; gw*_*ij*_ the interaction effect of the genotypic and environmental covariates in the *ij-th* genotype X environment combination which is modelled by the Hadamard product of $Z_{g} GZ_{g}^{T}$ and $\Omega$, denoted as $gw \sim N\left( {0, \left[ {Z_{g} GZ_{g}^{T} } \right] \circ \Omega \sigma_{gw}^{2} } \right)$ with *Z*_*g*_ as an incidence matrix for the vector of additive genetic effects.

The genomic heritability ($h_{g}^{2}$) of the studied traits was estimated based on the seven models described above. The different estimates of $h_{g}^{2}$ were obtained with the following formula (de los Campos et al. 2015):

$$h_{g}^{2} = \frac{{\sigma_{g}^{2} }}{{\sigma_{g}^{2} + \sigma_{\varepsilon }^{2} }}$$

where $\sigma_{g}^{2}$ is the additive genetic variance obtained with the genomic relationship matrix (G) and $\sigma_{\varepsilon }^{2}$ is the residual error variance as defined previously.

The analyses of genomic prediction were performed in R (R Core Team 2022) using the R statistical package *BGLR* (Pérez and de los Campos 2014). The hyperparameters for prior specification and the number of iterations for the Markov Chain Monte Carlo (MCMC) algorithm were set up with 25,000 iterations, with a burn-in of 5000 and a thinning of 10.

#### Cross-Validation Experiments: Assessing Predictive Abilities for Untested Environments

Three different cross-validations (CV) experiments were designed to assess the predictive abilities (PA) in untested environments. In the first CV experiment (CV-RAN), the target environment (validation set) was predicted using four environments selected randomly among the 14 remaining environments from the training set. Random sampling was repeated 50 times. The predictive ability was computed for each of the 50 replicates and then averaged. An ANOVA and Tukey's tests were then carried out at the significance level of 5% based on z-transformed values (Z = 0.5 [ln(1 + r) − ln(1 − r)]), to identify the significant differences in predictive ability (r) among the models in each environment. Analyses were performed separately for each trait. After the confidence limits and means for Z were estimated, these were transformed back to r values.

For the second CV experiment (CV-SEL), the target environment was predicted using four environments specifically selected among the remaining fourteen environments to form the training set. The selection of environments for the training set was based on Euclidean distance in terms of ECs. The closest environments were then identified (Additional file 1: Table S5). The prediction was performed once for each target environment.

For the third CV experiment, we used the “leave-one-environment-out" (CV-LOEO) method. The target environment was predicted using the remaining fourteen environments as a training set. Each environment was predicted using the model trained based on the information (genotypic and phenotypic data as well as ECs) of the remaining fourteen environments.

For the three CV experiments, the PAs were measured as the Pearson correlation coefficient between the predicted values and the adjusted phenotypes in the validation set (target environment).

#### Cross-Validation Experiment: Assessing Predictive Abilities for Untested Lines

For this CV experiment, we used the leave-one-out method for predicting the untested lines. The models were trained using all the environment and all the lines except one. The remaining line was predicted across all environments. We repeated this process for all 33 lines evaluated in all fifteen environments. In this CV experiment, the PAs (Pearson correlation coefficient) were measured in two ways: at the line level (correlation between the predicted values and the adjusted phenotypes across the fifteen environments for a given line) and at the environment level (correlation between the predicted values and the adjusted phenotypes in given environments across all the 33 lines).

The R scripts for the different CV experiments are provided in the Additional file 6.

## Results

### Characterization of Genetic Structure for the ECP

**Fig. 1:**
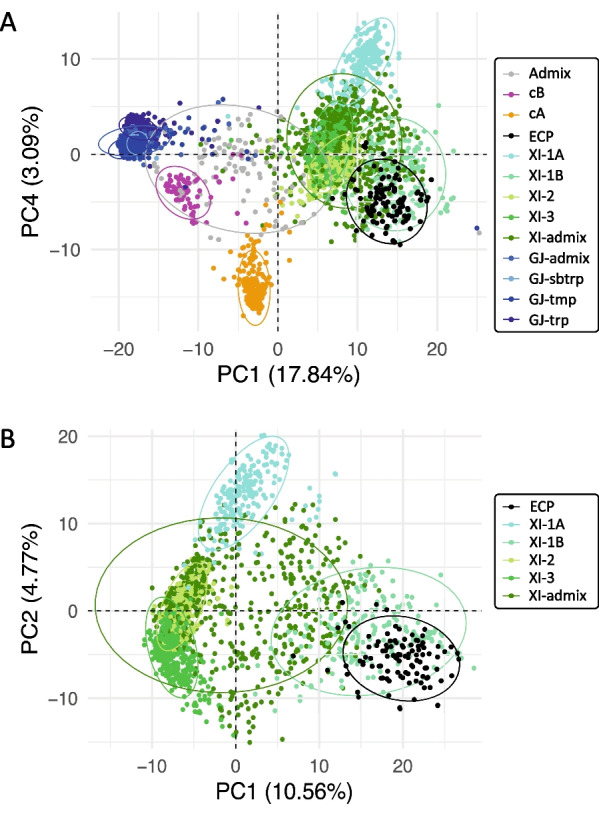
The principal component analysis between the elite core panel (ECP) and 3000 rice genomes (3K-RG) accessions. (**A**) The ECP with all subgroups of 3K-RG; (**B**) the ECP with only indica (XI) subgroups. The analysis is based on 837 common SNPs. The ECP lines are denoted with black dots. The subgroups from 3K-RG included Admix, circum-Basmati, circum-Aus, indica (1A, 1B, 2, 3, admix), and japonica (admix, subtropical, temperate, tropical)

The results showed that the Japonica (GJ), circum-Basmati (cB), circum-Aus (cA), and Indica (XI) subgroups from 3 K-RG were clearly separated and confirmed the clustering of the ECP into the XI subgroups (Fig. 1A). When only the Indica (XI) subgroups were used, the ECP was found to be close to the XI-1B subgroup (Fig. 1B). XI-1B is known to include essentially modern varieties largely generated by the IRRI’s breeding program in Southeast Asia. Similar results were found with the neighbor-joining tree between ECP with the whole 3 K-RG samples and with only the XI subgroups (Additional file 2: Fig. S4).

### Phenotypic Variation of the ECP Across Environments

**Fig. 2:**
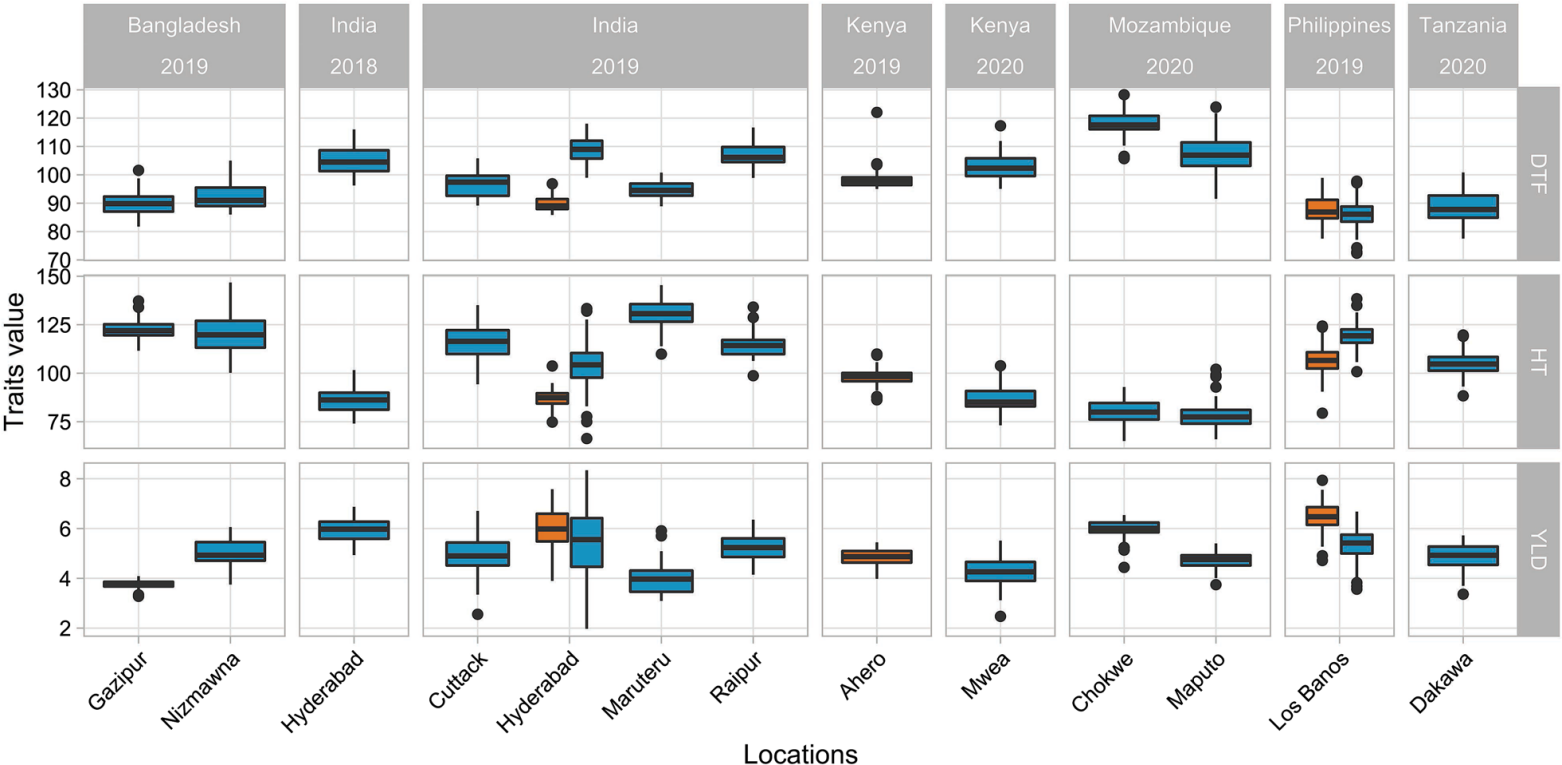
Distribution of phenotypic values of elite lines for the three traits evaluated across the 15 environments. *DTF* days to flowering; *HT* plant height; *YLD* grain yield. The boxes with orange colors indicated the trials conducted in the dry season, and the boxes with the blue color indicated the trials in the wet season

A large phenotypic variability was found for the three traits across all the environments (Table 2, Fig. 2). For DTF, the average value per trial ranged from 86 (Los Baños, wet season) to 118 days (Chokwe) with most of the trials displaying a vegetative phase of about 90 days. The duration of flowering (calculated as the difference between the earliest and latest in a given environment) ranged from 17 days (Hyderabad-dry season) to 56 days (Maputo) with an average value of 32 days. Trials at Ahero, Maputo, and Los Baños (dry and wet seasons) had longer flowering times compared to the others. For HT, a continuous gradient in the average value per trial was found with values ranging from 78.6 (Maputo) to 130.2 cm (Maruteru) (Table 2). As expected, a similar trend was observed for YLD with an average value per trial ranging from 3.76 (Gazipur) to 6.46 ton/ha (Los Baños-dry season).

**Table 2:** Phenotypic values and broad-sense heritability (H^2^) for the three traits across environments

Country	Location	Study name	DTF (days)		HT (cm)		YLD (t/ha)			H^2^ (*SE*)	
			Range	Mean	Range	Mean	Range	Mean	DTF	HT	YLD
Bangladesh	Gazipur	BD-GZ-19W	82–102	90	111.6–137.2	122.6	3.28–4.09	3.76	0.69 (1.02)	0.55 (2)	0.19 (0.15)
Bangladesh	Nizmawna	BD-NM-19W	86–105	92	100.2–146.8	120.4	3.75–6.06	5.01	–	–	–
India	Hyderabad	IN-HY-18W	96–116	105	74.1–101.6	85.9	4.93–6.87	5.94	0.85 (0.14)	0.74 (0.71)	0.67 (0.08)
India	Cuttack	IN-CU-19W	89–106	97	94.3–135.1	116.0	3.34–6.71	4.96	0.96 (0.02)	1 (0.0003)	0.89 (0.03)
India	Hyderabad	IN-HY-19D	86–97	90	74.8–103.7	87.1	3.90–7.58	6.01	0.64 (0.37)	0.72 (0.7)	0.7 (0.09)
India	Hyderabad	IN-HY-19W	99–118	109	75.0–133.3	104.5	1.97–8.34	5.47	–	–	–
India	Maruteru	IN-MA-19W	89–101	95	109.9–145.4	130.2	3.09–5.91	4.03	0.73 (0.41)	0.93 (0.15)	0.87 (0.05)
India	Raipur	IN-RP-19W	99–117	107	98.7–134.1	114.7	4.15–6.36	5.25	0.92 (0.08)	0.91 (0.29)	0.53 (0.19)
Kenya	Ahero	KE-AH-19D	95–104	98	86.3–109.9	98.1	3.98–5.45	4.83	0.52 (0.55)	0.51 (1.08)	0.28 (0.1)
Kenya	Mwea	KE-MW-20W	95–112	103	73.2–103.8	86.6	3.12–5.52	4.28	0.6 (0.65)	0.69 (0.84)	0.59 (0.09)
Mozambique	Chokwe	MZ-CK-20W	106–128	118	65.1–92.9	80.0	4.44–6.54	6.01	0.91 (0.04)	0.27 (6.81)	0.28 (0.1)
Mozambique	Maputo	MZ-MP-20W	92–124	108	66.0–102.1	78.6	3.74–5.40	4.70	0.78 (0.5)	0.7 (0.77)	0.45 (0.1)
Philippines	Los Baños	PH-LB-19D	78–99	88	90.5–124.3	107.2	4.72–7.94	6.46	0.87 (0.08)	0.73 (0.66)	0.75 (0.04)
Philippines	Los Baños	PH-LB-19W	72–98	86	100.8–138.4	119.3	3.56–6.68	5.33	0.82 (0.2)	0.5 (1.93)	0.71 (0.04)
Tanzania	Dakawa	TZ-DK-20W	78–101	88	88.4–119.7	104.9	3.36–5.72	4.87	0.93 (0.09)	0.74 (1.4)	0.89 (0.05)

*DTF* days to flowering; *HT* plant height; *YLD* grain yield. In the two environments of Bangladesh-Nizmawna and India-Hyderabad, the broad-sense heritability was not calculated due to the experimental design without replications

Broad-sense heritability (H^2^) was rather high for all three traits. The H^2^ ranged from 0.52 (Ahero) to 0.96 (Cuttack) for DTF, from 0.27 (Chokwe) to 1.0 (Cuttack) for HT, and from 0.19 (Gazipur) to 0.89 (Cuttack and Dakawa) for YLD trait (Table 2).

For most of the environments, the phenotypic correlations between traits (DTF, HT and YLD) were low to medium (− 0.31–0.53). No clear trend was identified for all environments, although HT was significantly correlated with days to flowering in nine of the environments and flowering was significantly correlated with yield in only five environments (Additional file 2: Fig. S5).

### *Characterization of G* × *E Interactions upon the Phenotypic Performance of ECP*

The environment, genotypes, and their interaction effects were found to be significant for the three traits (Additional file 1: Table S6). The heritabilities based on the combined analysis of all trials (h^2^_g_) confirmed the strong effect of the environments (Additional file 1: Table S7). For the models including the G × E interactions, h^2^_g_ ranged from 0.52 to 0.57 for DTH, from 0.41 to 0.44 for HT, and from 0.10 to 0.12 for YLD. The correlations between environments corroborated these differences between traits. For DTF, correlations with values ranging from 0.07 to 0.82 were found with 33% of the pairwise correlations greater than 0.5 (Additional file 2: Fig. S6A). A similar trend was observed for HT with correlations ranging from 0.04 to 0.77 and 54% of the correlation being greater than 0.5 (Additional file 2: Fig. S6B). On the contrary, only 18% of the correlations were significant in the case of YLD and most of the correlations were below 0.2 (Additional file 2: Fig. S6C). Three environments had significant correlations with most of the other environments for the three traits considered: Hyderabad-India (2018-WS), Mwea-Kenya and Los Baños (2019-DS).

**Fig. 3:**
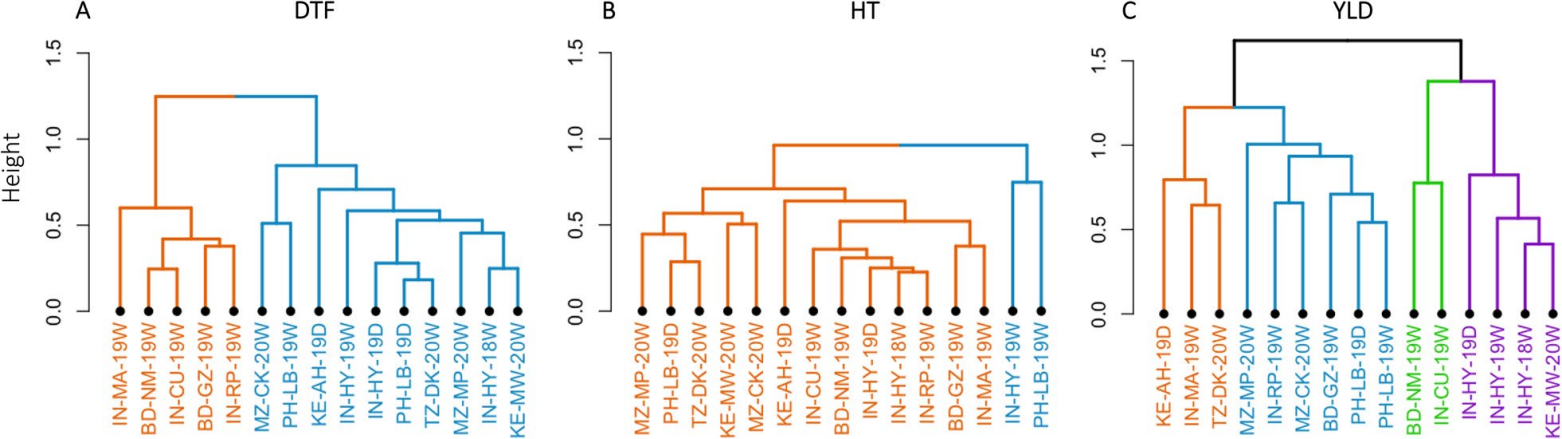
Hierarchical clustering of environments for the three traits. *DTF* days to flowering (panel **A**); *HT* plant height (panel **B**); *YLD* grain yield (panel **C**). The different colors present different clusters among environments. The names of environments in the clusters are formatted by ordering country name, location, year, and season (see Table 1)

In order to better identify similar environments based on phenotypic performances, a clustering analysis was conducted for the three traits (Fig. 3). For the DTF, two main clusters were identified: one comprising five locations from Bangladesh and India (except Hyderabad) and the other including ten locations from Africa, the Philippines and Hyderabad (Fig. 3A). For the HT, two main clusters were identified (Fig. 3B). The first cluster had only two environments (Hyderabad-wet season 2019 and Los Baños-wet season 2019) that presented lower correlations with other environments. The second cluster gathered all other environments. However, similarly to DTF, two subclusters tend to separate environments in Bangladesh and India to the rest (Africa and the Philippines). For YLD, since the level of correlation between environments was lower, the environments were spread in more clusters. Indeed, four clusters were identified with no clear structuration by regions or by seasons (Fig. 3C). However, the environments from the same location (Hyderabad or Los Baños) clustered together.

### Characterization of Environments Based on Environmental Covariates

**Fig. 4:**
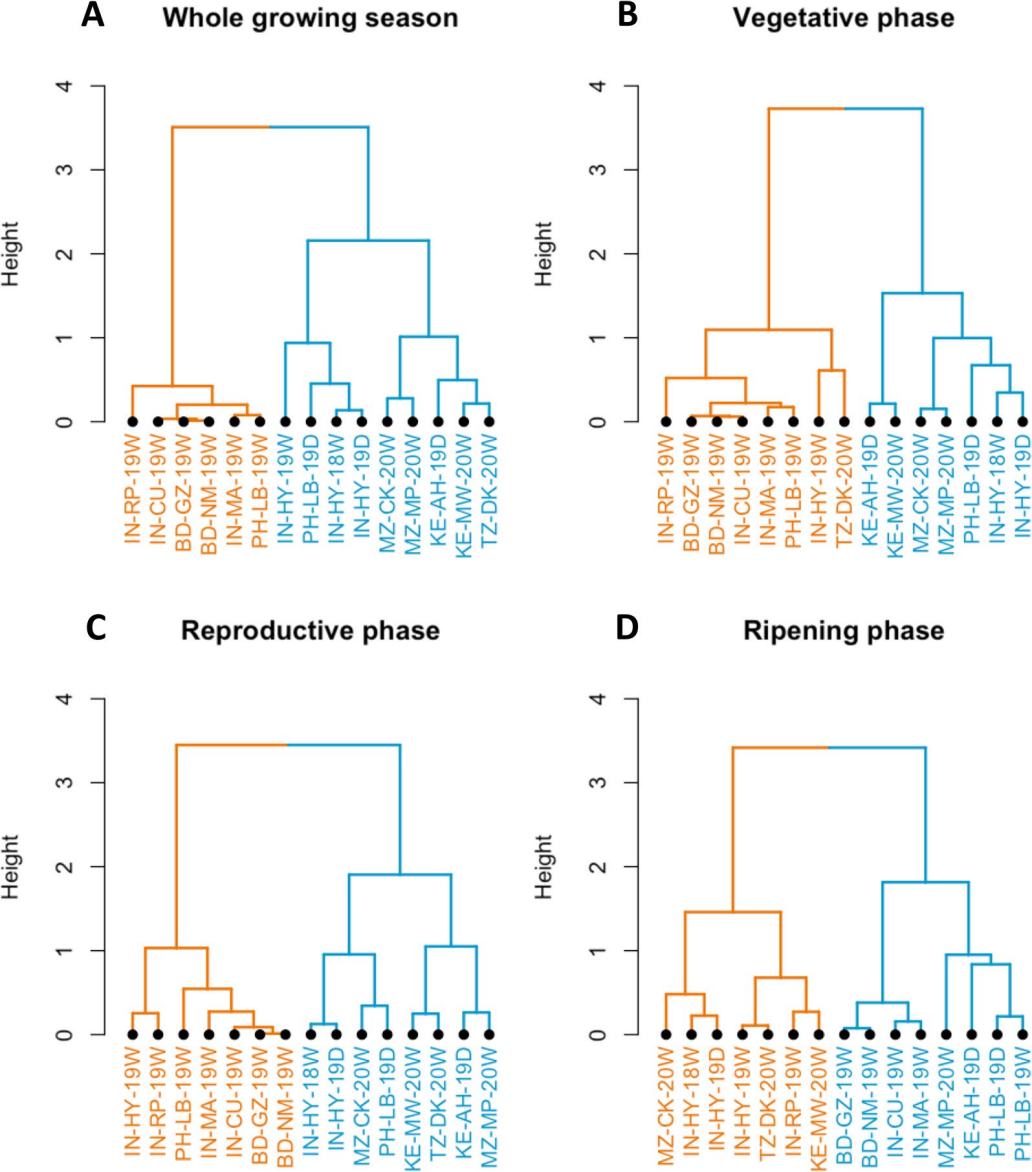
Hierarchical clustering of environments upon ECs throughout (**A**) the whole growing season, each developmental phase (**B**–**D**). Different colors show the different clustering between environments. The names of environments in the clusters are formatted by ordering country name, location, year, and season (see Table 1)

The clustering analysis for the four periods showed different patterns (Fig. 4A–D). For the whole growing season, two main clusters were found. The first cluster grouped India's, and Bangladesh's environments and Los Baños in the wet season. The second cluster included a subcluster of Hyderabad’s environments and Los Baños in the dry season, and a subcluster with all of Africa’s environments. Similar results were found for the reproductive phase with two main clusters. These clusters were also similar to the clustering of environments for DTF but very different from those of HT and YLD traits. For the vegetative and ripening phases, environments from Asia tend to cluster with environments from Africa with no clear separation between the two regions.

### Genomic Prediction for Untested Environments

#### Impact of the Prediction Models

**Fig. 5:**
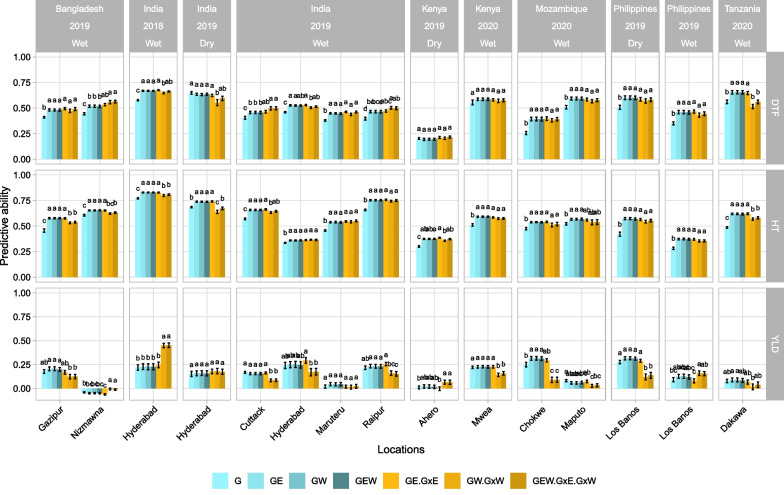
Predictive abilities for untested environments using the CV-RAN scenario. Seven different models are compared (see material and methods section). The letters at the top of each bar represent the results of Tukey’s HSD comparison between models in each environment. The means between two groups are significantly different (*p*-value < 0.05) if there is no letter in common. The error bars are presented by PA mean ± SE where SE is the standard error of PA values from 50 replicates. *DTF* days to flowering; *HT* plant height; *YLD* grain yield

We evaluated the efficiency of the different models to predict untested environments with the CV-RAN scenario. The predictive abilities (PAs) ranged from 0.19 (Ahero) to 0.67 (Hyderabad-2018) for DTF, from 0.28 (Los Baños - wet season) to 0.83 (Hyderabad-2018) for HT and from − 0.06 (Nimwa) to 0.45 (Hyderabad-2018) for YLD (Additional file 1: Table S8). As expected, DTH and HT presented higher PAs than YLD. Considering the models, the integration of the main effect of the environment (E) or the environmental covariates (W) significantly increased the PA for DTF (12 environments over 15) and HT (all environments) compared to baseline model G (Fig. 5). However, for YLD, the GE, GW and GEW models did not perform significantly better than the G model, except in one case (Chokwe). Interestingly, in most of the cases, no significant increase in PA was found between models including the interaction term (G × E or/and G × W) and GE, GW or GEW model. Indeed, for DTF, the models with interactions were significantly better than models with main effects in only three environments. For HT and YLD, the models with interactions (more specifically with GxW) showed a significant decrease in PA in five and six environments, respectively.

#### Impact of Training Set Composition

**Fig. 6:**
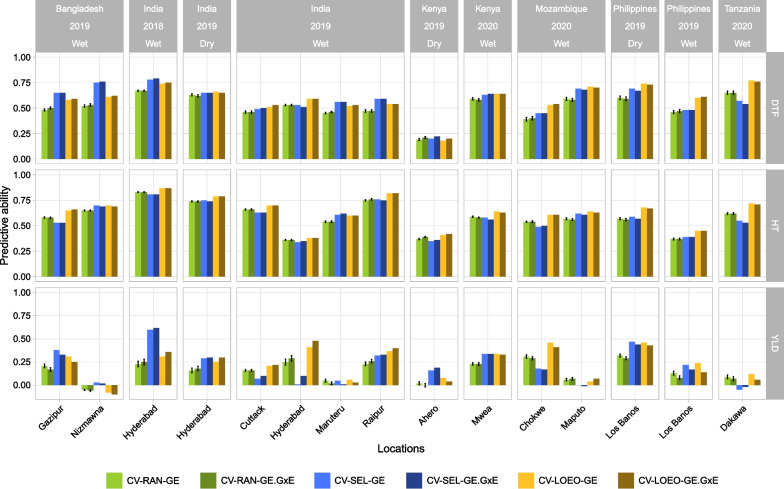
Comparison of the predictive abilities between the three cross-validation scenarios: *CV-RAN* (random), *CV-SEL* (selected environments) and *CV-LOEO* (leave one environment out). Two models are presented: the first one with only the main effects of genotypes and environments (GE) and the second one with the main effect and the interaction (GE − G × E). The error bars are presented by PA mean ± SE where SE is the standard error of PA values from 50 replicates. *DTF* days to flowering; *HT* plant height; *YLD* grain yield

We compared three cross-validation scenarios (CV-RAN, CV-SEL, and CV-LOEO) with only GE and GE-G × E models, to evaluate the effect of training set composition on PA. For DTF, the PA ranged from 0.19 (Ahero) to 0.67 (Hyderabad 2018) for CV-RAN, from 0.2 (Ahero) to 0.79 (Hyderabad 2018) for CV-SEL, and from 0.18 (Ahero) to 0.77 (Dakawa) for CV-LOEO. For HT, PA varied from 0.36 (Hyderabad 2019 wet season) to 0.83 (Hyderabad 2018), from 0.34 (Hyderabad 2019 wet season) to 0.81 (Hyderabad 2018) and from 0.38 (Hyderabad 2019 wet season) to 0.87 (Hyderabad 2018) for CV-RAN, CV-SEL and CV-LOEO, respectively. While YLD reached PA ranging from − 0.06 (Nizmawna) to 0.32 (Los Baños 2019 dry season), from − 0.05 (Dakawa) to 0.62 (Hyderabad 2018) and from − 0.1 (Nizmawna) to 0.48 (Hyderabad 2019 wet season) for CV-RAN, CV-SEL and, CV-LOEO, respectively (Fig. 6). The CV-LOEO scenario presented the highest PAs in five to fourteen environments depending on the trait. The CV-SEL scenario was the second in terms of PA with higher PA in two to six environments. Similar results were found when comparing CV scenarios using GW and GW − G × W models, or GEW and GEW − G × E–G × W models (Additional file 2: Fig. S7A–B). To see the impact of the experimental design, we calculated PAs with CV-SEL and CV-LOEO using a subset of 33 common lines in all environments. The results revealed similar trends in PA between balanced and unbalanced datasets: no major gain in PA was observed when the interactions were included in the models (Additional file 1: Table S9–S10).

### Genomic Prediction for Untested Lines

The performances of untested lines across the fifteen environments were predicted with high accuracy with all the models including environmental effects or environmental covariates (GE, GW, GEW, GE-GxE, GW-GxW and GEW-GxE-GxW models, Table 3). The PAs were close to 0.95 on average for DTF and HT, and close to 0.81 on average for YLD. No differences were found between these models. As expected, the model with only the main effect of the genotypes (G) displayed PA close to 0 on average. When we looked at the environment level, we found lower PAs and large differences between environments. The PA ranged from − 0.03 to 0.56 for DTF, − 0.05 to 0.53 for HT and − 0.37 to 0.52 for YLD. A similar trend to that of the untested environment prediction was found: the models with the main effects of the environment (GE, GW, and GEW) tend to present higher PA than the other models. However, a large variability was found between environments and traits (Additional file 1: Table S11).

**Table 3:** Predictive abilities of untested lines

Model	DTF			HT			YLD		
	Range	Mean	SE	Range	Mean	SE	Range	Mean	SE
G	− 0.59–0.53	− 0.10	0.056	− 0.48–0.47	− 0.01	0.046	− 0.46–0.37	− 0.05	0.039
GE	0.82–0.99	0.94	0.007	0.8–0.99	0.95	0.008	0.4–0.94	0.79	0.021
GW	0.82–0.99	0.94	0.007	0.8–0.99	0.95	0.008	0.39–0.94	0.79	0.021
GEW	0.82–0.99	0.94	0.007	0.8–0.99	0.95	0.008	0.4–0.94	0.79	0.021
GE-GxE	0.8–0.99	0.95	0.007	0.79–0.99	0.94	0.009	0.57–0.95	0.81	0.018
GW-GxW	0.79–0.99	0.95	0.008	0.8–0.99	0.94	0.008	0.43–0.95	0.79	0.019
GEW-GxE-GxW	0.79–0.99	0.95	0.008	0.79–0.99	0.94	0.009	0.58–0.95	0.81	0.018

*DTF* days to flowering; *HT* plant height; *YLD* grain yield; *SE* standard error values

The average values are computed from predictive ability across 33 lines in each model

## Discussion

### Performance of Elite Breeding Lines

The characterization at both genetic and phenotypic levels of elite lines is a key aspect of breeding programs. This information allows the breeder to drive the breeding population in the desired direction while making efficient use of the available genetic diversity. In the framework of IRRI breeding programs for irrigated systems, a panel representing the elite diversity of the program was constituted in 2018 and then enriched with recent parental lines (Juma et al. 2021). In this study, we took advantage of the ECP and evaluated it in 15 environments in Asia and Africa. Although the trials were conducted with the standard practices for irrigated systems, important differences in the average performances were found between environments for three traits measured (DTF, HT, YLD). For example, a difference of 32 days was found for DTF between the two extreme environments. For YLD, the productivity was on average 2.7 t.ha higher in the most productive environment compared to the least productive. In addition to these differences, our results showed medium G × E for DTF and HT and a strong G × E for YLD. These levels of G × E are slightly higher than the ones generally found in similar studies on rice (Monteverde et al. 2019; Morais Júnior et al. 2017; Spindel et al. 2015). These results can partly be explained by the wide distribution of the trials and the associated environmental variations. Indeed, the clustering analysis based on eight ECs and four different phases (whole growing season, vegetative, reproductive, and ripening phases) showed similarity to the ones based on phenotypic performances. However, the clustering structure did not clearly separate Asian and African environments. This information will be used for a better definition of the target population of environments in the future (Atlin et al. 2000).

### Prediction Accuracies of Multi-environment Models

In rice, a wide variety of populations (diversity panels, breeding population, biparental crosses,…) have been used in GS studies depending on the context and the objective (Bartholomé et al. 2022). In the present study, we focused our efforts on a set of elite breeding materials phenotyped by the partners of the program. The number of environments available enables us to assess the impact of G × E modeling on PA for untested environments and untested lines using seven genomic prediction models. For untested environments, the approach resulted in high PAs for different combinations of trait/environment with values as high as 0.77 for DTF, 0.88 for PH, and 0.62 for YLD. However, YLD was poorly predicted in nearly half of the environments with values close to zero. This difference between more heritable traits (e.g. DTF and HT) and less heritable traits (e.g. YLD) has been already reported in the literature on rice (Ben Hassen et al. 2018; Monteverde et al. 2018; Morais Júnior et al. 2017). For untested lines, the PAs were very high (0.80–0.90) highlighting the complexity of predicting performance in new environments versus predicting new lines in known environments. We also found that, in most cases, the integration of environments (E), environmental covariates (W), and interaction effects (G × E or/and G × W components) increased PA when compared to the baseline G model. Interestingly, the integration of the interaction effects did not result in better PAs for all environments and in some cases even decreased the PA, especially for HT and YLD. We found a similar trend with a smaller but balanced data set suggesting that the poor estimation of the G × E was related to other factors such as the use of reaction norms to model the interactions (Cuevas et al. 2016). In rice, two studies reported the use of multi-environment models to predict the performances of genotypes in untested environments and obtained similar results (Monteverde et al. 2019; Morais Júnior et al. 2017). Morais Júnior et al. (2017) used historical data from three cycles of a breeding program with a total of 10 environments to assess the predictive ability of a single-step reaction norm model. They obtained high accuracies for the prediction of untested environment for the three traits evaluated: DTF (0.5–0.9), HT (0.25–0.7), and YLD (0.15–0.65). Morais Júnior et al. (2017) also evaluated the effect of GxE modeling in the context of the prediction of untested lines but did not find important differences with the models including only the main effects. Using two breeding populations (*indica* and *japonica*), Monteverde et al. (2019) found that modeling the interaction effects with the G × W component (G + W + G × W) did not give better results than modeling the main effects of genotypes and ECs (G + W). Similarly to our results, the integration of the interactions (G × W) even decreases the PA in some cases compared to the simple GBLUP model (G model). These results contrast with previous studies on barley and wheat where the use of ECs to model the environmental effects has resulted in higher prediction accuracies for untested environments (Jarquín et al. 2014; Malosetti et al. 2016). Previous studies on rice also showed that the modelling of G × E interactions tends to increase PA (Baertschi et al. 2021; Ben Hassen et al. 2018; Bhandari et al. 2019; Monteverde et al. 2018). However, most of these studies predict the performance of untested lines in known environments using two common cross-validation approaches to evaluate the PA of multi-environment models: CV1 and CV2 (Burgueño et al. 2012). For example, Ben Hassen et al. (2018) reported a better prediction performance of multi-environment models than single environment models using a diversity panel phenotyped under alternate wetting and drying and continuous flooding conditions. The gain in accuracy of multi-environment models over single-environment models was 30% under CV2. Similar results were also reported by Monteverde et al. (2018) and Baertschi et al. (2021) but with contrasted gains depending on the traits.

### Impact of Training Set Composition

To achieve a higher level of PA for untested environments, the selection of environments to compose the training set can play an important role (Jarquín et al. 2014). In this study, the PAs were found to be higher for both CV-LOEO (all environments) and CV-SEL (four correlated environments) compared to the CV-RAN (four random environments), confirming that using a training set with only correlated environments can be a good strategy. Indeed, several studies have shown that correlations between environments is a key factor in achieving good prediction accuracy, and the use of training data derived from correlated environments can improve prediction accuracy (Spindel and McCouch 2016). For example, Rogers and Holland (2022), using empirical data on maize, found a sharp decrease in predictive ability for the scenario “leave out related environments” compared to the scenario “leave out related hybrids”. They concluded that environmental similarity is an important driver of prediction accuracy compared to genetic similarities for environment-specific predictions. In a study on rice, Spindel et al. (2016) found that one of the major differences in prediction accuracies was associated with the level of correlation between environments, in which the prediction accuracies were generally higher when the training data used were from well-correlated environments. In this context, the use of environmental covariates is central to guide the choice of phenotyping sites and potentially reduce phenotyping efforts while maintaining a high level of precision. Therefore, the topic of multi-environmental prediction models and integration of ECs has gradually developed over the past decade in the plant breeding community (Crossa et al. 2022). In contrast to optimizing the composition of the training set (genotypes), optimizing the environmental information to be used for training the models has received less attention (Isidro et al. 2015; Rio et al. 2021).

### Implications for the Breeding Strategy at IRRI

Much of the complexity of plant breeding programs arises from G × E. For traits with a large proportion of G × E, such as yield, breeders have different options for evaluating them in their breeding programs. Since the costs of phenotyping are usually a major limitation, a small number of promising genotypes are evaluated in multi-environment trials to quantify the level of G × E and select the genotypes with the best performance (Comstock 1977). This can be a limitation if the goal is to exploit G × E interactions rather than minimize them. For this reason, the concept of a target population of environments (TPE) was defined. This is a set of environments that are homogeneous in terms of phenotypic perforations in which future varieties will be grown (Crespo-Herrera et al. 2021). However, it can be difficult to sample efficiently the TPE, especially in small public breeding programs. Being able to predict the performance of untested environments using multi-environment models and ECs can be very useful for a breeding program that operates in different countries like the IRRI program for irrigated systems. Recently, the program was redesigned to integrate genomic selection with enhanced multi-environment evaluations (first-stage yield trials) with the partners. The objective was to shorten the breeding cycle while optimizing multi-environment evaluations (Bartholomé et al. 2022). The findings of the present study support the idea to use all the phenotypic information from correlated environments to make the prediction. Currently, the predictions are made by region but the results from the CV-SEL and CV-LOEO showed that information from other environments can be borrowed to increase the PA. In practice, the use of ECs can help to consider more carefully the correlations between the different environments and therefore restructure the genomic prediction pipeline. In addition, perhaps the program is currently implementing a sparse-testing approach that aims to increase the number of lines evaluated while keeping the number of plots to a manageable size (Atanda et al. 2021; Jarquin et al. 2020). In that context, the genotypes are not fully replicated across environments making the estimation of GxE interactions more difficult. It is, therefore, necessary to go towards the estimation of the marker by environment interactions or marker by ECs to keep maintain the level of accuracy.

## Conclusion

Understanding the level of G × E in a given population and a given set of environments or locations is essential to better guide the testing strategy of a breeding program. However, the number of environments that can be evaluated by a program is often limited. The use of genomic prediction can be useful in a different way in this aspect. In this study, we showed that multi-environment models can predict untested lines with high accuracy. However, the prediction of an untested environment presents some challenges. We showed that models with only the main effects (G + E or G + W) were sufficient to obtain a good level of accuracy and that modelling the genotype by environment interaction (G × E or G × W) did not increase the accuracy. These results will allow more efficient use of the information generated by the IRRI breeding program and optimization of the testing strategy for updating the GS models.

## Supplementary Information

**Additional file 1**: **Table S1**. Composition of the elite core panel (ECP) used in this study. **Table S2**. Information on each field trial's locations and technical parameters. **Table S3**. Description of the statistical models used for phenotypic data analysis. **Table S4**. Environmental covariates (ECs) of each environment throughout the whole growing season for the different phases. vegetative (VE), reproductive (RE), and ripening (RI). **Table S5**. List of environments selected for the CV-SEL scenario. **Table S6**. Analysis of variance (ANOVA) for the three traits. **Table S7**. Genomic heritability (h²g) and the associated standard error (SD) for the three traits: days to flowering (DTF), plant height (PH) and grain yield (YLD). **Table S8**. The predictive abilities for the DTF, HT, and YLD traits for the CV-RAN scenario. **Table S9**. The predictive abilities for the DTF, HT, and YLD traits for the CV-SEL scenario. **Table S10**. The predictive abilities for the DTF, HT, and YLD traits for the CV-LOEO scenario. **Table S11**. Predictive abilities for each environment, based on the predictions of untested lines.

**Additional file 2**: **Fig. S1**. Physical positions of the 882 SNPs on the 12 chromosomes of rice using Nipponbare reference genome. **Fig. S2**. Graphical representation of the allocation of ECP across 15 environments. Blue vertical lines represent the ECP-environment combinations that were observed, and the white lines (blanks) correspond to unobserved combinations. **Fig. S3**. Schematic representation of the developmental stages of ECP in each environment. **Fig. S4**. Unweighted neighbor-joining trees between the ECP and *Oryza sativa* groups from 3K-RG (A), or the indica subgroups (B). **Fig. S5**. The scatter plot, histogram, and correlation between the DTF, HT, and YLD traits in the single environments. **Fig. S6**. The scatter plot, histogram, and correlation between environments for DTF trait (A), HT trait (B) and YLD trait (C). **Fig. S7**. Comparison of predictive abilities between the three cross-validation scenarios. (A) models with genotypes and environmental covariates' main effects (GW) and their interactions (GW-GxW); (B) models combined all main effects (GEW) and their interactions (GEW-GxE-GxW).

**Additional file 3**: The genotypic data of elite-breeding lines (882 SNPs).

**Additional file 4**: The phenotypic data (BLUPs values) of three traits of elite-breeding lines across 15 environments.

**Additional file 5**: Environmental covariates (ECs) of each environment throughout the whole growing season for the different phases: vegetative (VE), reproductive (RE), and ripening (RI).

**Additional file 6**: R scripts to perform the genomic prediction analysis.

## Data Availability

The datasets analyzed during the current study are included in this published article and its additional files.

## Abbreviations

ECP: Elite core panel
GS: Genomic selection
GBLUP: Genomic best linear unbiased prediction
PA: Predictive ability
CV: Cross-validation
