# Supplementary material for: Multi-environment Genomic Selection in Rice Elite Breeding Lines
Source: Rice (N Y). 2023 Feb 8;16:7. doi: 10.1186/s12284-023-00623-6 (PMC9908796; doi:10.1186/s12284-023-00623-6)
Supplement: Supplementary file 2 — Additional file 2: Fig. S1. Physical positions of the 882 SNPs on the 12 chromosomes of rice using Nipponbare reference genome. Fig. S2. Graphical representation of the allocation of ECP across 15 environments. Blue vertical lines represent the ECP-environment combinations that were observed, and the white lines (blanks) correspond to unobserved combinations. Fig. S3. Schematic representation of the developmental stages of ECP in each environment. Fig. S4. Unweighted neighbor-joining trees between the ECP and Oryza sativa groups from 3K-RG (A), or the indica subgroups (B). Fig. S5. The scatter plot, histogram, and correlation between the DTF, HT, and YLD traits in the single environments. Fig. S6. The scatter plot, histogram, and correlation between environments for DTF trait (A), HT trait (B) and YLD trait (C). Fig. S7. Comparison of predictive abilities between the three cross-validation scenarios. (A) models with genotypes and environmental covariates' main effects (GW) and their interactions (GW-GxW); (B) models combined all main effects (GEW) and their interactions (GEW-GxE-GxW). [file 12284_2023_623_MOESM2_ESM.pdf]

Supplementary information

Multi-environment genomic selection in rice elite breeding lines

Nguyen V. H.[1,2,3], Morantte R. I. Z.[3], Lopena V.[3], Verdeprado H.[3], Murori R.[3], Ndayiragije A.[3], Katiyar S. [3], Islam M. R. [3], Juma R.[3], Galvez H.[4], Glaszmann J-C.[1,2], Cobb J. N. [3,5], Bartholomé J.[2,6,7*]

1 CIRAD, UMR AGAP Institut, F-34398, Montpellier, France

2 UMR AGAP Institut, Univ Montpellier, CIRAD, INRAE, Institut Agro, Montpellier, France

3 Rice Breeding Platform, International Rice Research Institute, DAPO Box7777, Metro Manila, Philippines

4 University of the Philippines Los Baños, College, Los Baños, Laguna 4031, Philippines

5 RiceTec. Inc, PO Box 1305, Alvin, TX 77512

6 CIRAD, UMR AGAP Institut, Cali, Colombia

7 Alliance Bioversity-CIAT, Cali, Colombia

*Corresponding author: jerome.bartholome@cirad.fr

This additional file is containing the supplementary figures from the main text of the paper

Fig. S1 Physical positions of the 882 SNPs on the 12 chromosomes of rice using Nipponbare reference genome.

Fig. S2 Graphical representation of the allocation of ECP across 15 environments. Blue vertical lines represent the ECP-environment combinations that were observed, and the white lines (blanks) correspond to unobserved combinations.

Fig. S3 Schematic representation of the developmental stages of ECP in each environment. The main three developmental stages of each environment were identified in the following: the vegetative stage: the interval time from the transplanting date up to the date of 50% flowering of the first ECP; the flowering stage: being the interval time between the first ECP and the last ECP are flowered of 50%; maturity stage: the interval time from the date of 50% flowering of the last ECP up to harvesting.

Fig. S4 Unweighted neighbor-joining trees between the ECP and Oryza sativa groups from 3K-RG (A), or the indica subgroups (B). The NJ trees were constructed using 837 common SNPs by combining the SNPs data of 3K-RG (Wang et al., 2018) and the ECP from the 1K-RiCA assay.

Fig. S5 The scatter plot, histogram, and correlation between the DTF, HT, and YLD traits in the single environments. The names of environments are abbreviated with the format of country-location-year and season. At BD (Bangladesh), the trial environments were conducted at GZ (Gazipur), and NM (Nizmawna). At IN (India) conducted at HY (Hyderabad), CU (Cuttack), MA (Maruteru), and RP (Raipur). At KE (Kenya) conducted at AH (Ahero), and MW (Mwea). At MZ (Mozambique) conducted at CK (Chokwe), and MP (Maputo). At PH (Philippines) conducted at LB (Los Banos). At TZ (Tanzania) conducted at DK (Dakawa). 18W: Wet season in 2018. 19D: Dry season in 2019. 19W: Wet season in 2019. 20W: Wet season in 2020. *, **, ***: statistically significant at 5% ($p<0.05$), 1% ($p<0.01$), and 0.1% ($p<0.001$).

Fig. S6 The scatter plot, histogram, and correlation between environments for DTF trait (A), HT trait (B) and YLD trait (C). The names of environments are abbreviated with the format of country-location-year and season. At BD (Bangladesh), the trial environments were conducted at GZ (Gazipur), and NM (Nizmawna). At IN (India) conducted at HY (Hyderabad), CU (Cuttack), MA (Maruteru), and RP (Raipur). At KE (Kenya) conducted at AH (Ahero), and MW (Mwea). At MZ (Mozambique) conducted at CK (Chokwe), and MP (Maputo). At PH (Philippines) conducted at LB (Los Banos). At TZ (Tanzania) conducted at DK (Dakawa). 18W: Wet season in 2018. 19D: Dry season in 2019. 19W: Wet season in 2019. 20W: Wet season in 2020. *, **, ***: statistically significant at 5% ($p<0.05$), 1% ($p<0.01$), and 0.1% ($p<0.001$).

Fig. S7 Comparison of predictive abilities between the three cross-validation scenarios. (A) models with genotypes and environmental covariates main effects (GW) and their interactions (GW-GxW); (B) models combined all main effects (GEW) and their interactions (GEW-GxE-GxW).

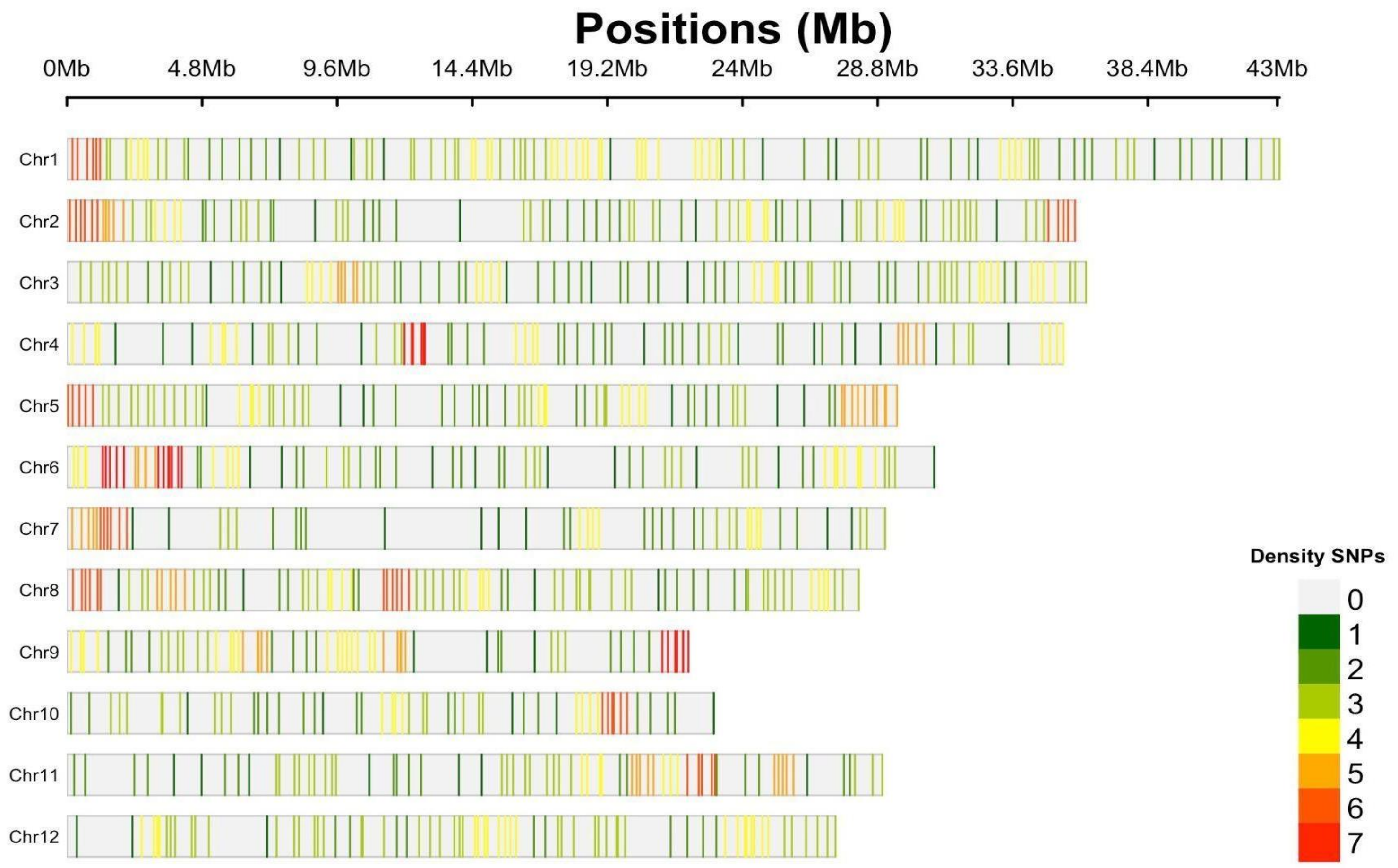

Fig. S1 Physical positions of the 882 SNPs on the 12 chromosomes of rice using Nipponbare reference genome.

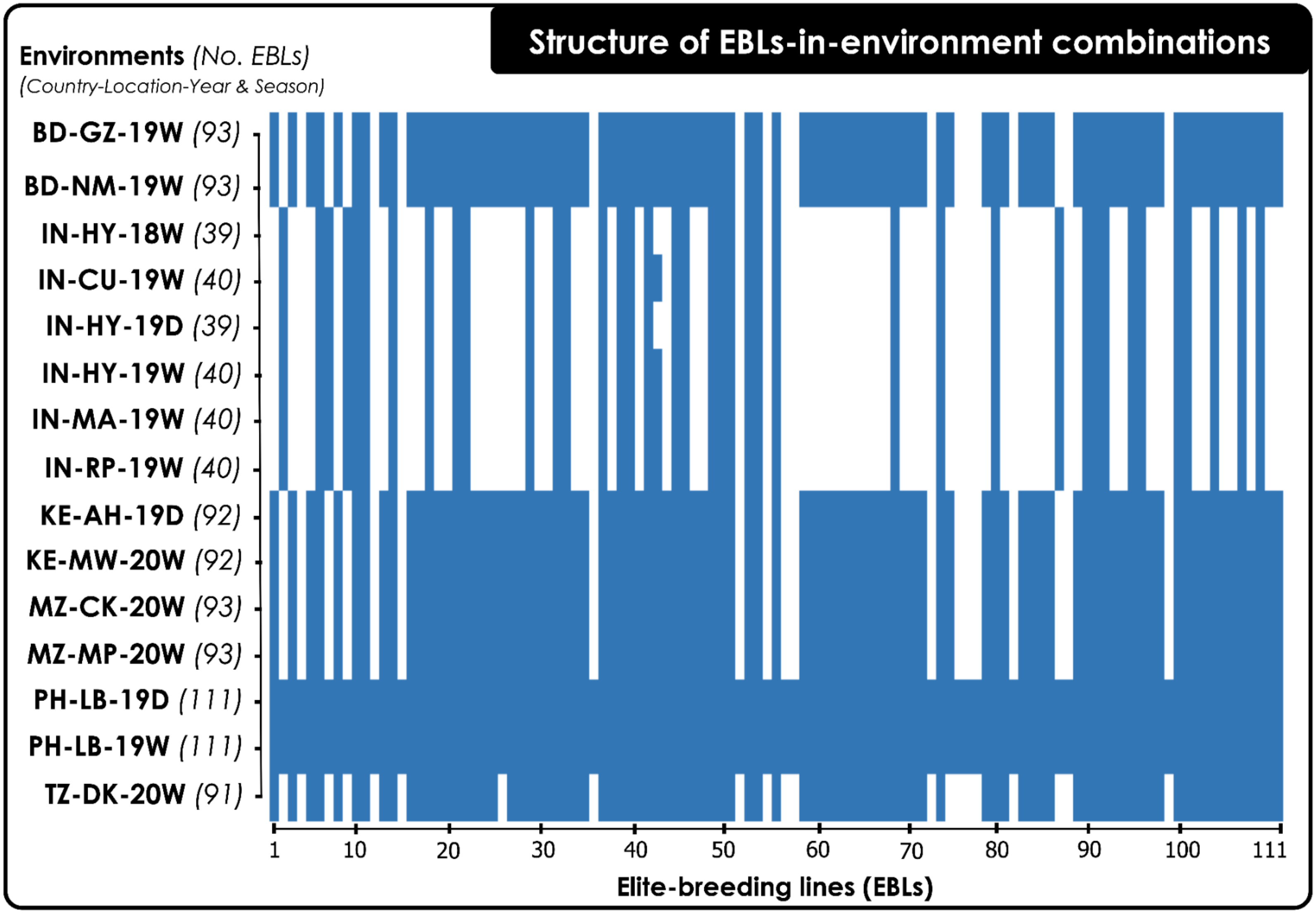

Fig. S2 Graphical representation of the allocation of ECP across 15 environments. Blue vertical lines represent the ECP-environment combinations that were observed, and the white lines (blanks) correspond to unobserved combinations.

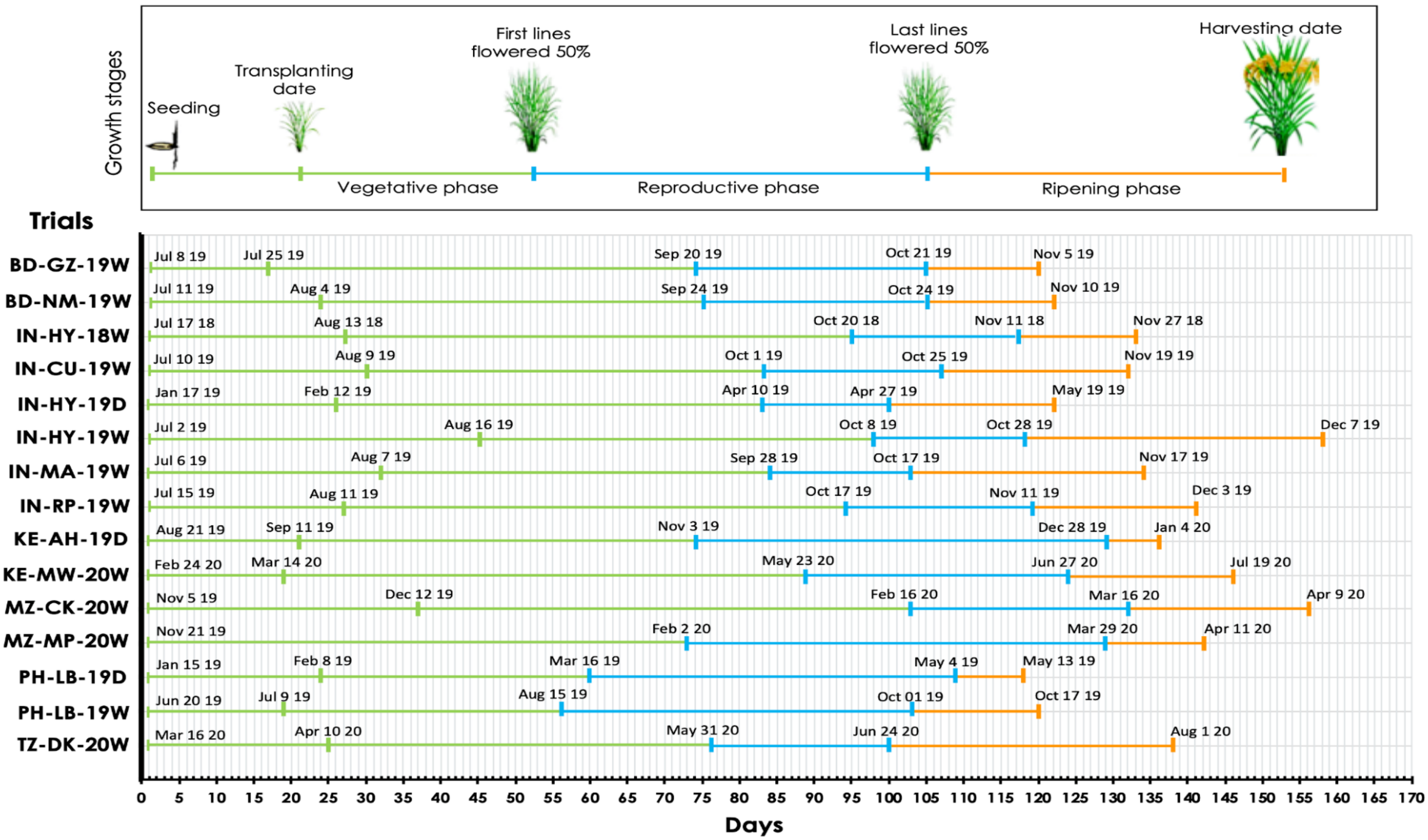

Fig. S3 Schematic representation of the developmental stages of ECP in each environment. The main three developmental stages of each environment were identified in the following: the vegetative stage: the interval time from the transplanting date up to the date of 50% flowering of the first ECP; the flowering stage: being the interval time between the first ECP and the last ECP are flowered of 50%; maturity stage: the interval time from the date of 50% flowering of the last ECP up to harvesting.

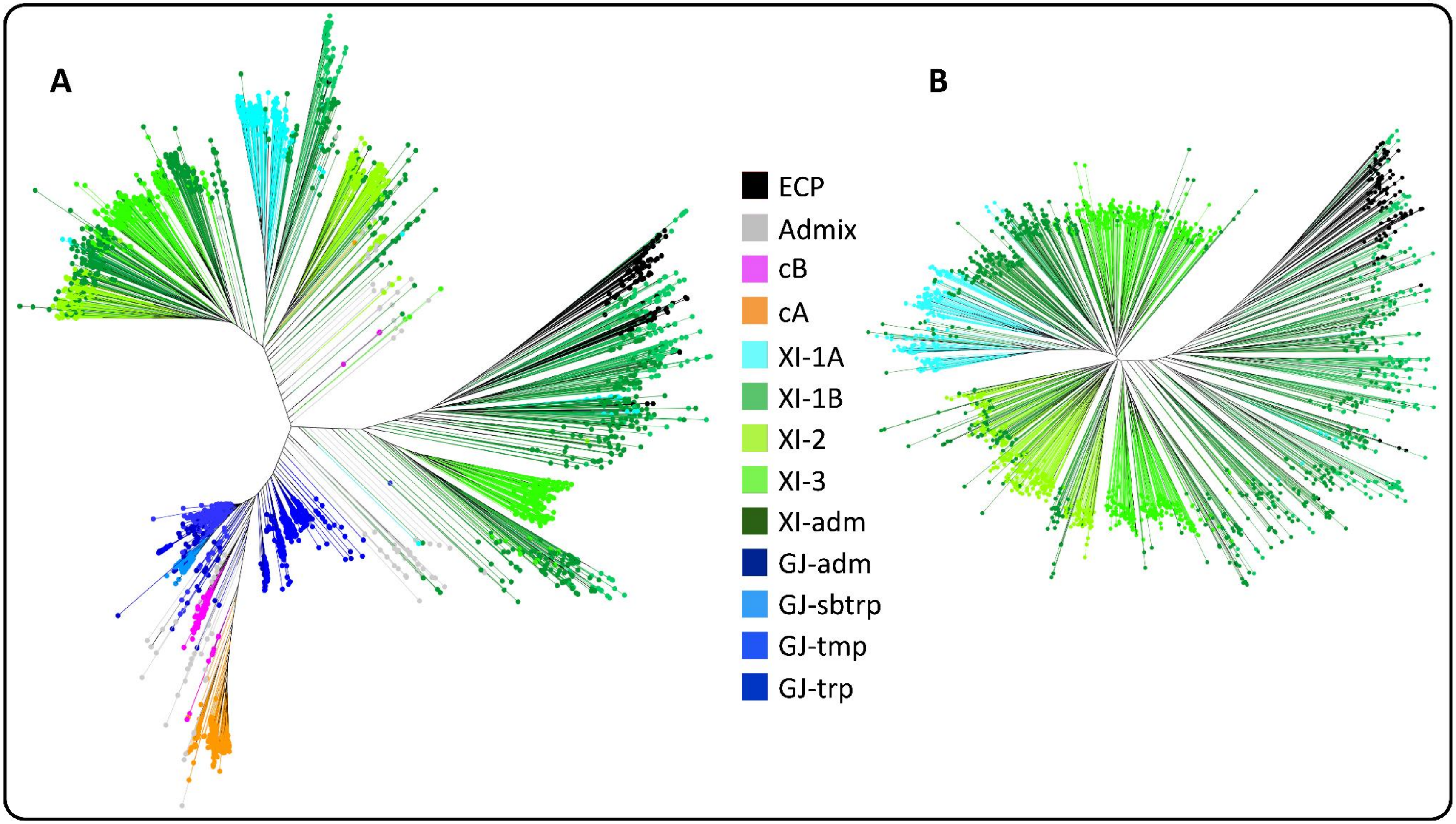

Fig. S4 Unweighted neighbor-joining trees between the ECP and *Oryza sativa* groups from 3K-RG (A), or the *indica* subgroups (B). The NJ trees were constructed using 837 common SNPs by combining the SNPs data of 3K-RG (Wang *et al.*, 2018) and the ECP from the 1K-RiCA assay.

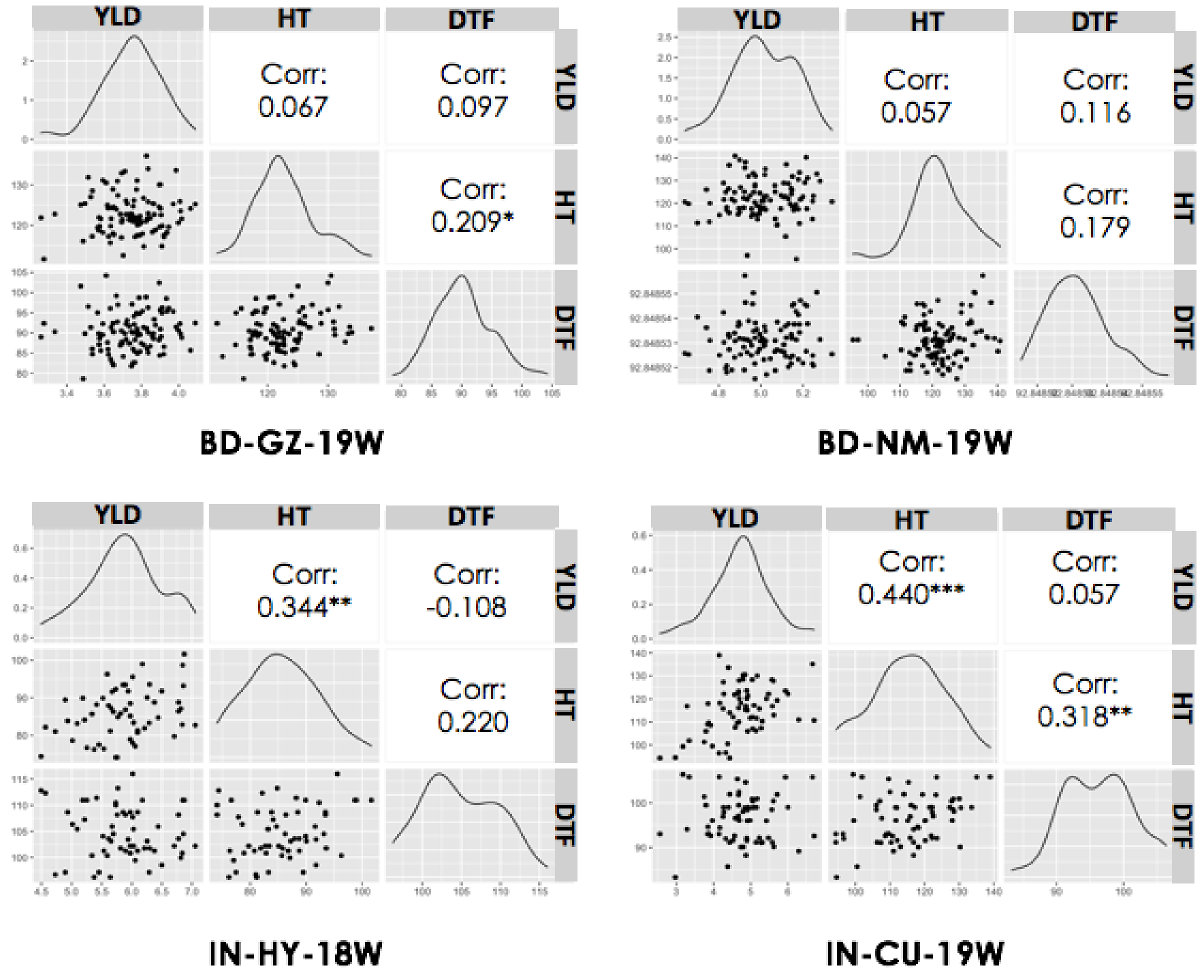

Fig. S5 The scatter plot, histogram, and correlation between the DTF, HT, and YLD traits in the single environments. The names of environments are abbreviated with the format of country-location-year and season. At BD (Bangladesh), the trial environments were conducted at GZ (Gazipur), and NM (Nizmawna). At IN (India) conducted at HY (Hyderabad), CU (Cuttack), MA (Maruteru), and RP (Raipur). At KE (Kenya) conducted at AH (Ahero), and MW (Mwea). At MZ (Mozambique) conducted at CK (Chokwe), and MP (Maputo). At PH (Philippines) conducted at LB (Los Banos). At TZ (Tanzania) conducted at DK (Dakawa). 18W: Wet season in 2018. 19D: Dry season in 2019. 19W: Wet season in 2019. 20W: Wet season in 2020. *, **, ***: statistically significant at 5% ($p<0.05$), 1% ($p<0.01$), and 0.1% ($p<0.001$).

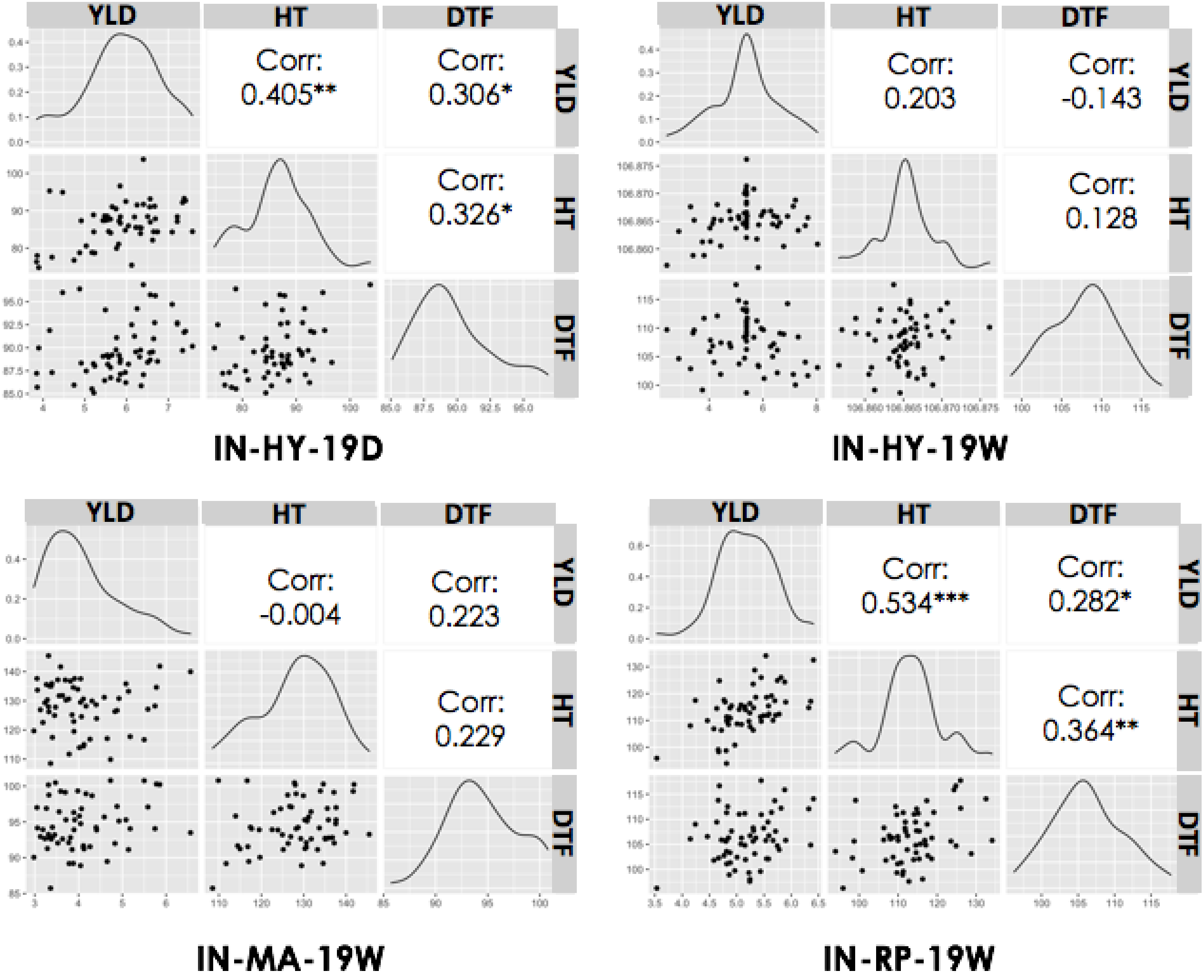

Fig. S5 Continued.

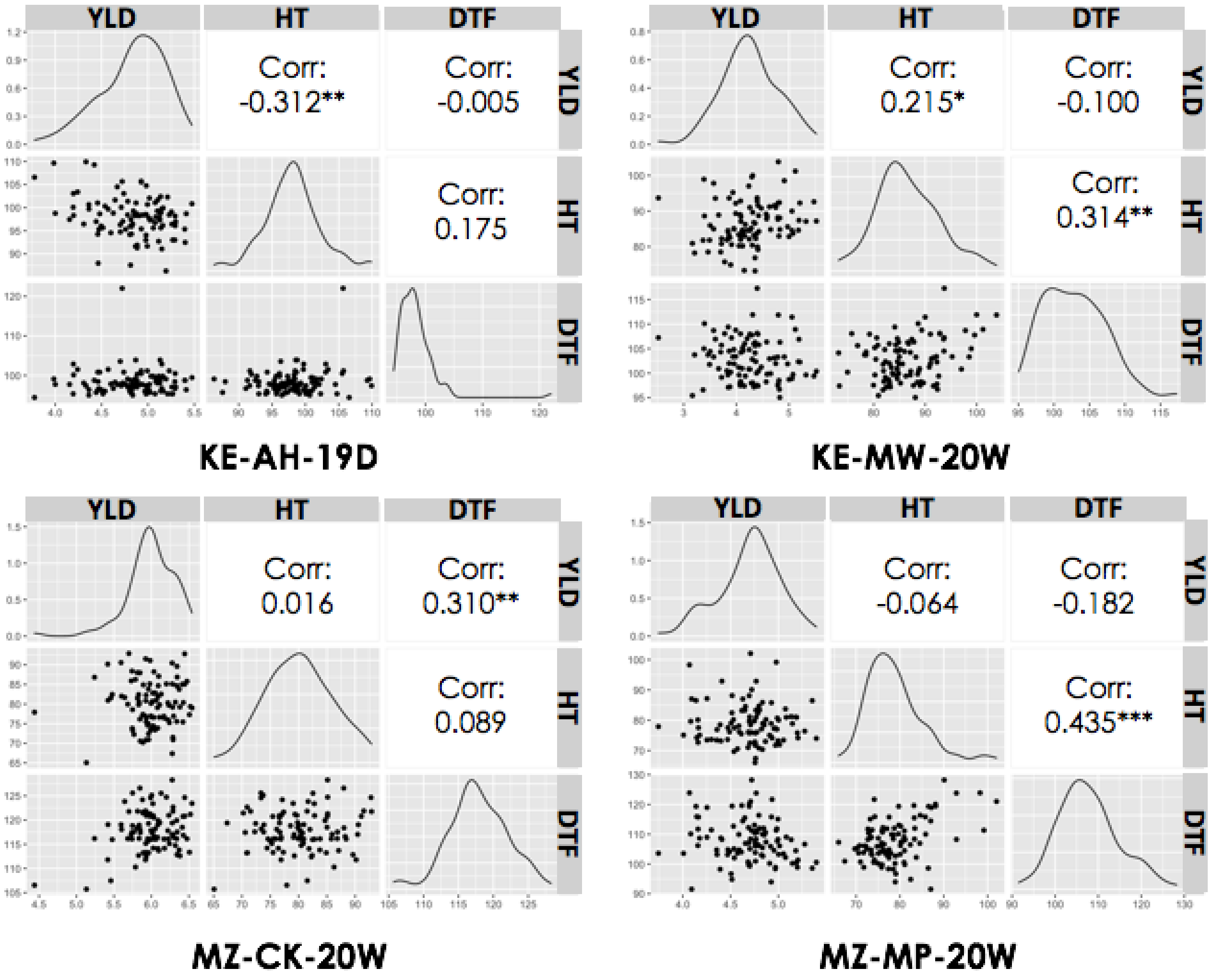

Fig. S5 Continued.

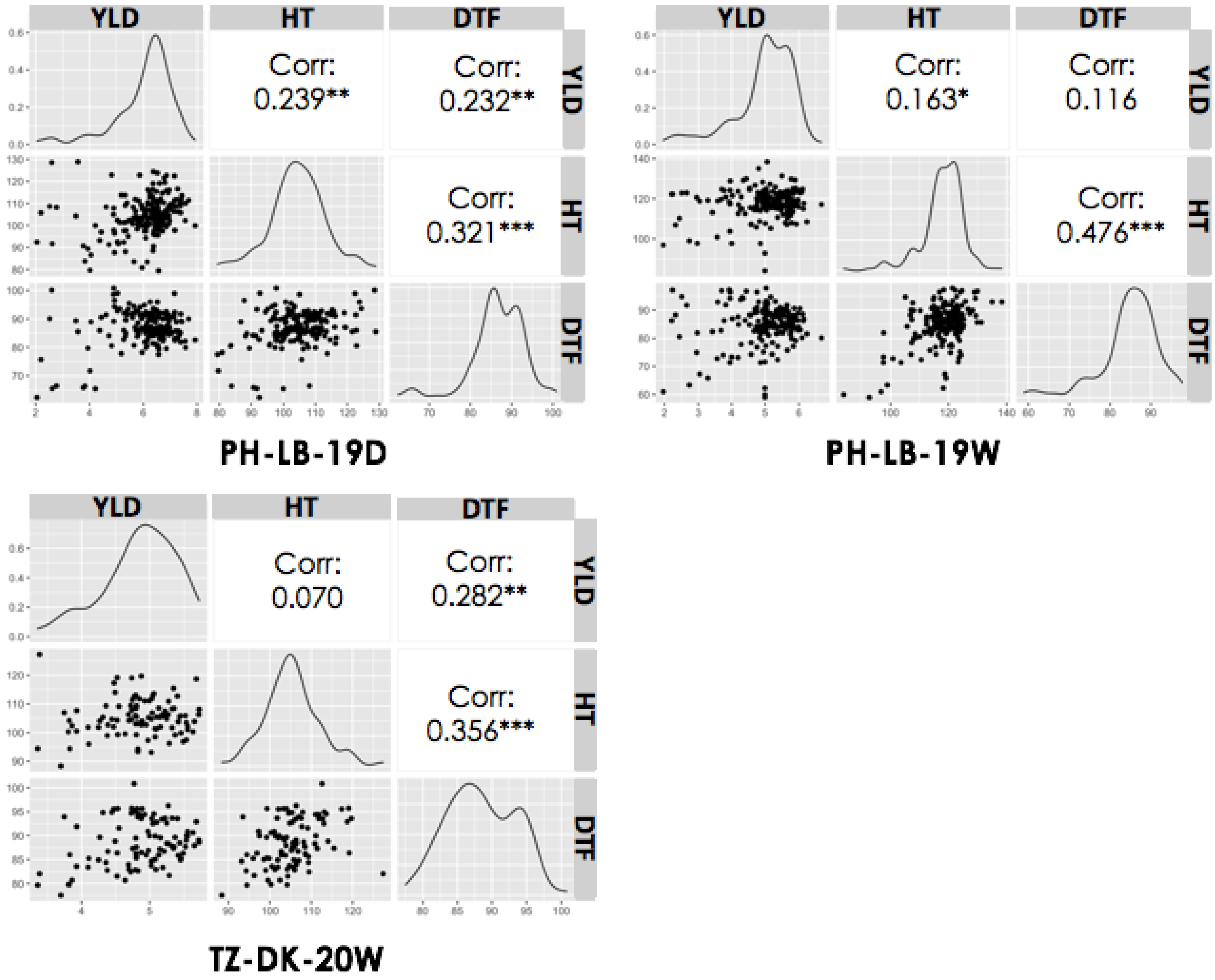

Fig. S5 Continued.

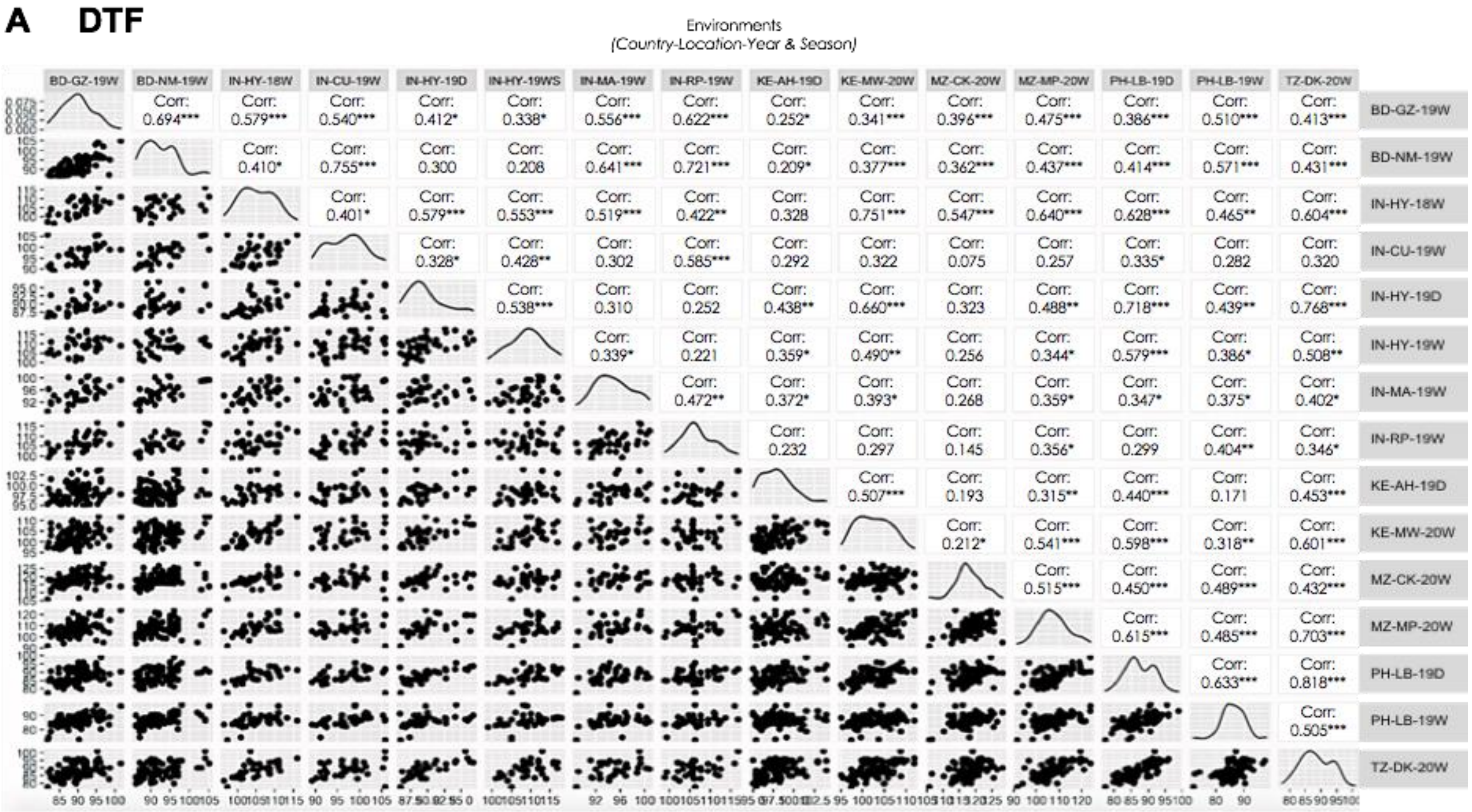

Fig. S6 The scatter plot, histogram, and correlation between environments for DTF trait (A), HT trait (B) and YLD trait (C). The names of environments are abbreviated with the format of country-location-year and season. At BD (Bangladesh), the trial environments were conducted at GZ (Gazipur), and NM (Nizmawna). At IN (India) conducted at HY (Hyderabad), CU (Cuttack), MA (Maruteru), and RP (Raipur). At KE (Kenya) conducted at AH (Ahero), and MW (Mwea). At MZ (Mozambique) conducted at CK (Chokwe), and MP (Maputo). At PH (Philippines) conducted at LB (Los Banos). At TZ (Tanzania) conducted at DK (Dakawa). 18W: Wet season in 2018.

19D: Dry season in 2019. 19W: Wet season in 2019. 20W: Wet season in 2020. *, **, ***: statistically significant at 5% (p<0.05), 1% (p<0.01), and 0.1% (p<0.001).

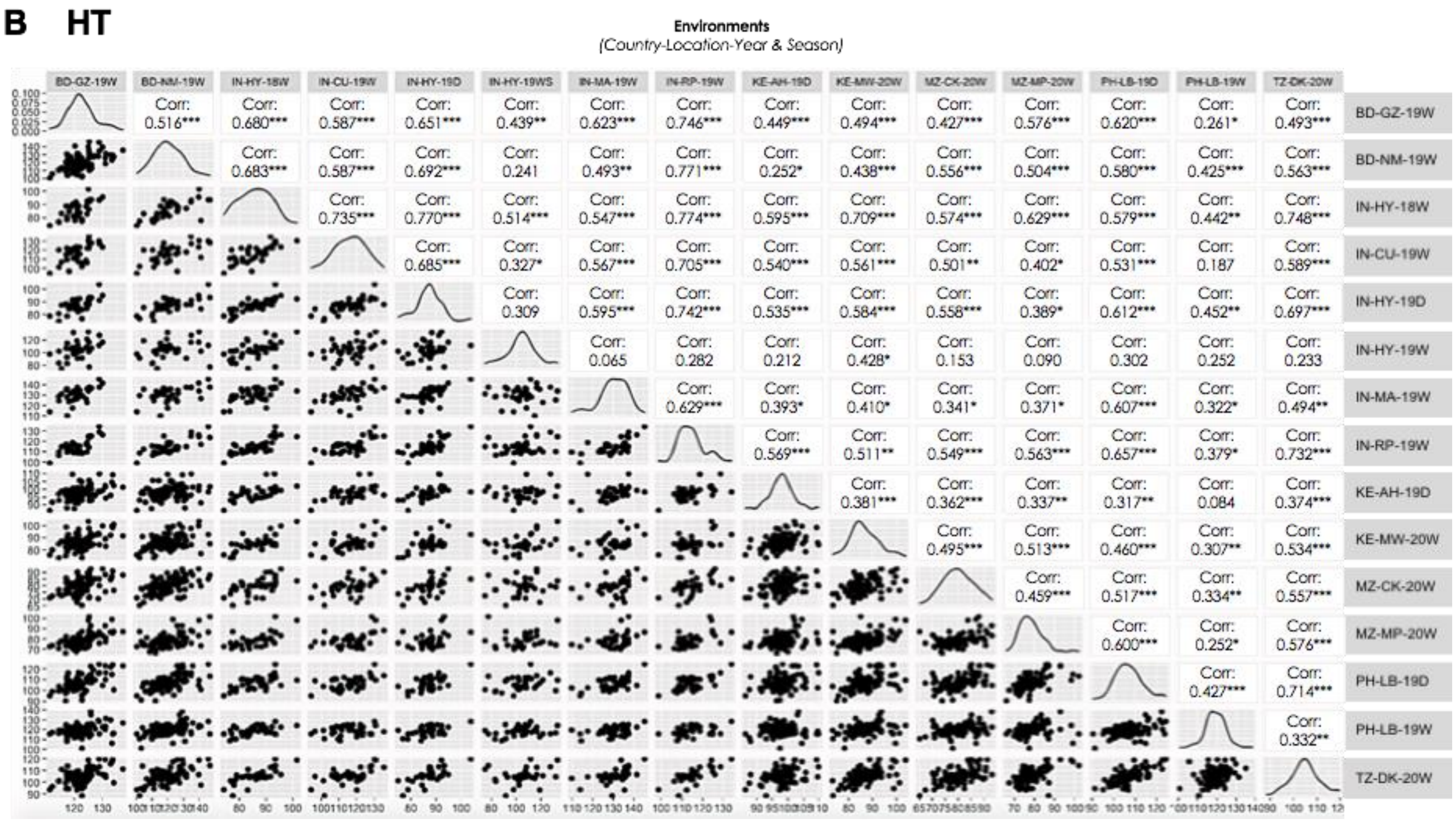

Fig. S6 Continued.

C YLD

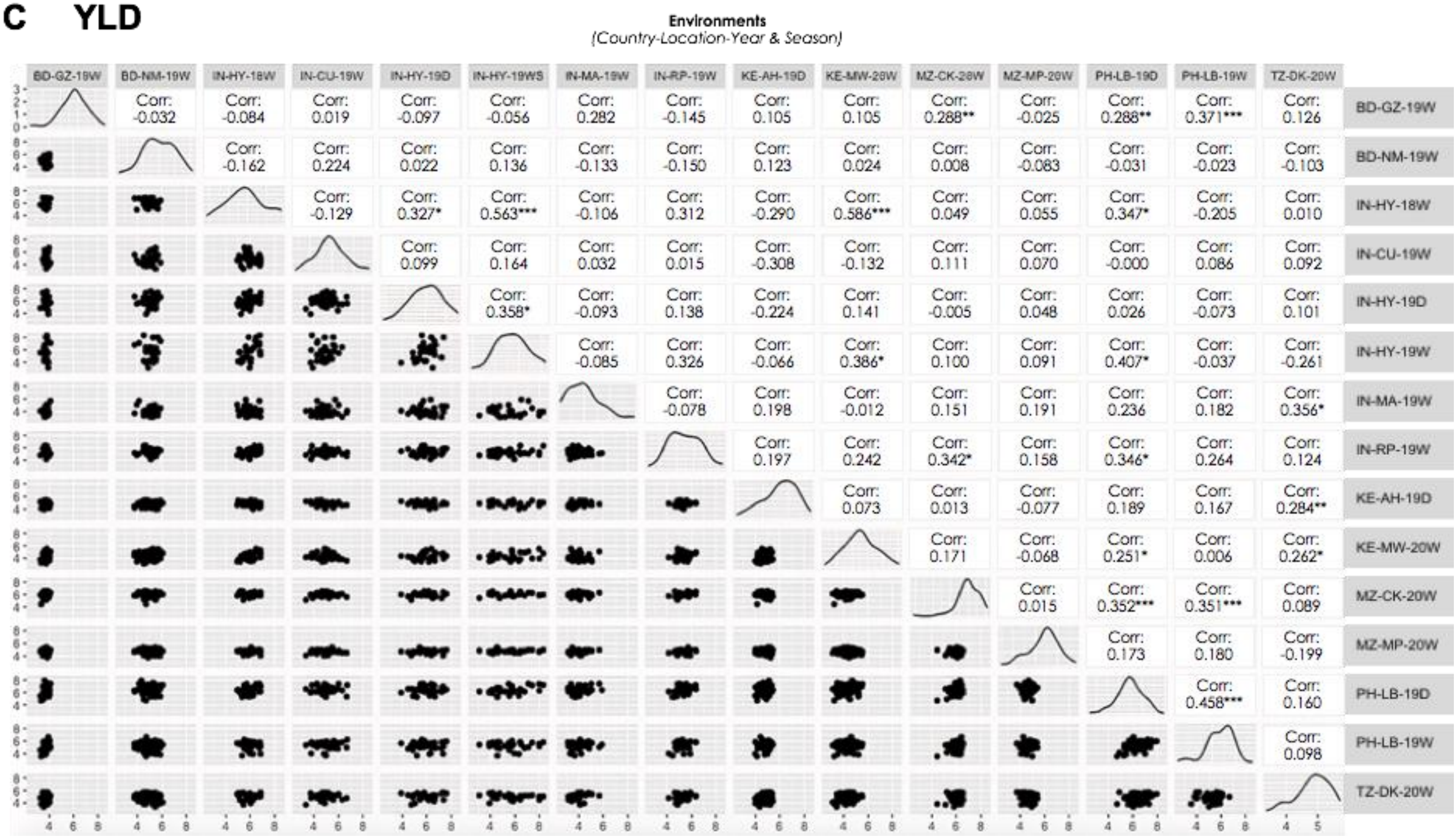

Fig. S6 Continued.

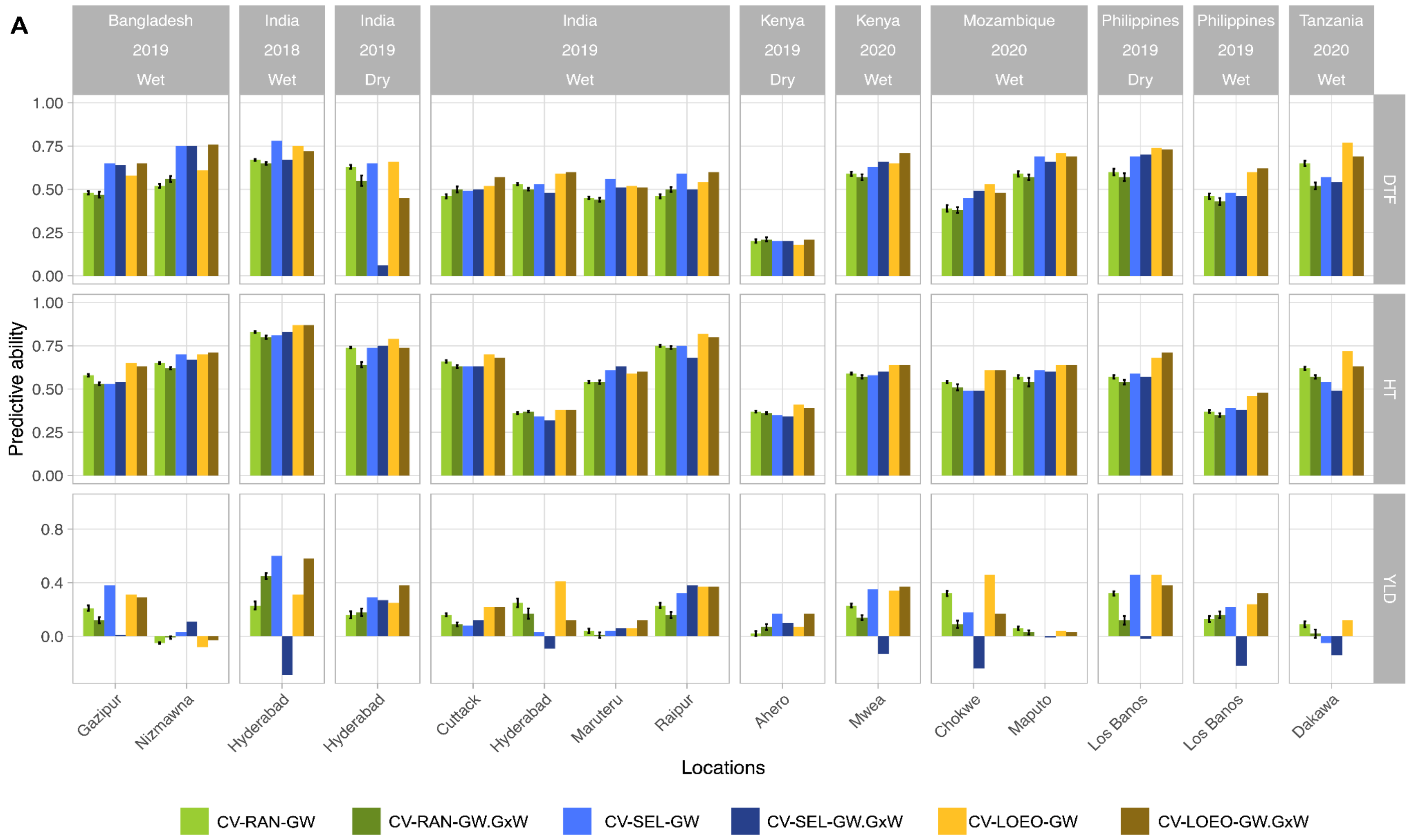

Fig. S7 Comparison of predictive abilities between the three cross-validation scenarios. (A) models with genotypes and environmental covariates main effects (GW) and their interactions (GW-GxW); (B) models combined all main effects (GEW) and their interactions (GEW-GxE-GxW).

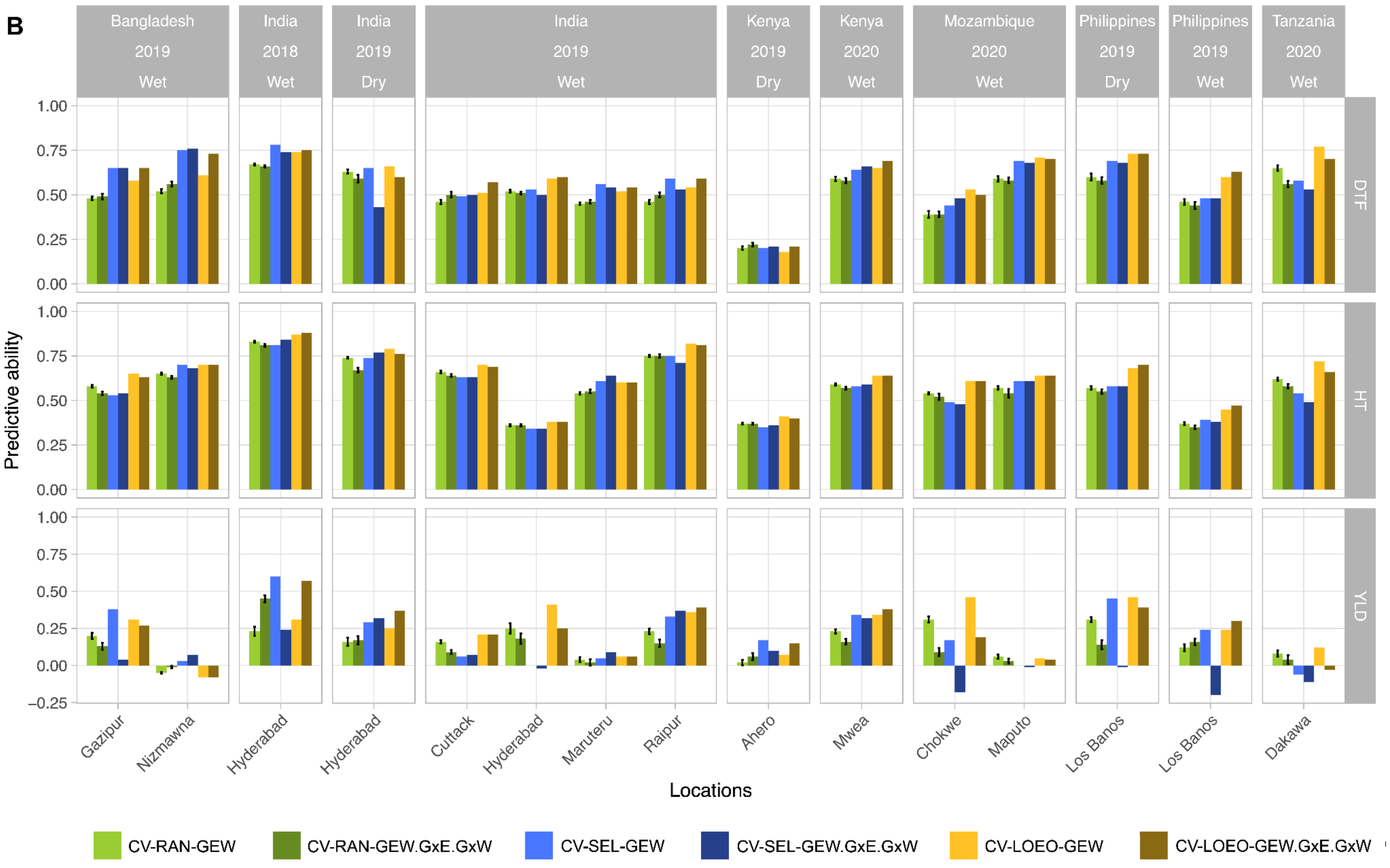

Fig. S7 Continued.
